# Multiple types of data are required to identify the mechanisms influencing the spatial expansion of melanoma cell colonies

**DOI:** 10.1186/1752-0509-7-137

**Published:** 2013-12-12

**Authors:** Katrina K Treloar, Matthew J Simpson, Parvathi Haridas, Kerry J Manton, David I Leavesley, DL Sean McElwain, Ruth E Baker

**Affiliations:** 1 Mathematical Sciences, Queensland University of Technology, Brisbane, Australia; 2 Tissue Repair and Regeneration Program, Institute of Health and Biomedical Innovation, Queensland University of Technology, Brisbane, Australia; 3 Centre for Mathematical Biology, Mathematical Institute, Radcliffe Observatory Quarter, Woodstock Road, Oxford, OX2 6GG, UK

**Keywords:** Melanoma, Cancer, Cell migration, Cell proliferation, Cell–to–cell adhesion, Circular barrier assay, Mathematical model

## Abstract

**Background:**

The expansion of cell colonies is driven by a delicate balance of several mechanisms including cell motility, cell–to–cell adhesion and cell proliferation. New approaches that can be used to independently identify and quantify the role of each mechanism will help us understand how each mechanism contributes to the expansion process. Standard mathematical modelling approaches to describe such cell colony expansion typically neglect cell–to–cell adhesion, despite the fact that cell–to-cell adhesion is thought to play an important role.

**Results:**

We use a combined experimental and mathematical modelling approach to determine the cell diffusivity, *D*, cell–to–cell adhesion strength, *q*, and cell proliferation rate, *λ*, in an expanding colony of MM127 melanoma cells. Using a circular barrier assay, we extract several types of experimental data and use a mathematical model to independently estimate *D*, *q* and *λ*. In our first set of experiments, we suppress cell proliferation and analyse three different types of data to estimate *D* and *q*. We find that standard types of data, such as the area enclosed by the leading edge of the expanding colony and more detailed cell density profiles throughout the expanding colony, does not provide sufficient information to uniquely identify *D* and *q*. We find that additional data relating to the degree of cell–to–cell clustering is required to provide independent estimates of *q*, and in turn *D*. In our second set of experiments, where proliferation is not suppressed, we use data describing temporal changes in cell density to determine the cell proliferation rate. In summary, we find that our experiments are best described using the range *D*=161−243*μ*m^2^hour^−1^, *q*=0.3−0.5 (low to moderate strength) and *λ*=0.0305−0.0398hour^−1^, and with these parameters we can accurately predict the temporal variations in the spatial extent and cell density profile throughout the expanding melanoma cell colony.

**Conclusions:**

Our systematic approach to identify the cell diffusivity, cell–to–cell adhesion strength and cell proliferation rate highlights the importance of integrating multiple types of data to accurately quantify the factors influencing the spatial expansion of melanoma cell colonies.

## Background

Cell colony expansion is driven by several mechanisms including cell motility, cell–to–cell adhesion and cell proliferation [1-3]. Methods that can be used to quantify the role of these various mechanisms driving *in vitro* colony expansion will assist in improving our understanding of them [2,4-7]. In this work, we propose a systematic approach to identify and quantify the mechanisms driving the expansion of melanoma cell colonies *in vitro*.

We choose to study melanoma cells since melanoma is the most dangerous form of skin cancer, which can spread rapidly and cause serious illness and death [8-10]. Approximately 75% of all skin cancer deaths are due to melanoma, and each year 132,000 new cases are diagnosed globally, with more than 12,500 of these reported in Australia [11]. While the five–year survival rate in patients with non–metastatic melanoma can be as high as 95%, the five–year survival rate for patients with metastatic melanoma is less than 15% [12]. The precise details of the mechanisms that drive melanoma cell colony expansion are unclear, and using a systematic approach which can independently identify and quantify the role of each individual mechanism may provide practical insights into how colonies of melanoma cells expand [4,13-15].

Expanding colonies of cells are characterised by moving cell fronts [1,16], and typical mathematical modelling approaches to describe the movement of such fronts use partial differential equations that incorporate descriptions of cell motility and cell proliferation [1,2,7,16-18]. In most cases, the terms describing cell motility and cell proliferation in the partial differential equation are chosen without explicitly considering the details of the underlying biological process [1,19,20], and often neglect cell–to–cell adhesion [21-23]. However, several experimental studies have observed that the loss of cell–to–cell adhesion between individual melanoma cells is associated with increased spatial expansion [24-29], suggesting that cell–to–cell adhesion plays an important role in the spatial expansion of melanoma cell colonies.

An alternative modelling approach to describe the expansion of cell colonies involves simulating the behaviour of individual cells in a colony in a discrete modelling framework [30-39]. Discrete models have the benefit that they produce data, such as snapshots and movies, that are more compatible with experimental data compared to partial differential equation models [7]. Furthermore, discrete models can be designed to incorporate realistic cell behaviours which can be more difficult using a partial differential equation description [7]. A recent view of discrete cell–based modelling approaches can be found in [38,40,41]. Khain *et al.*[42,43] developed a discrete mathematical model to describe the expansion of a motile and proliferative cell colony in which the cell motility is reduced by cell–to–cell adhesion. In their model, they represented simulated cells on a two–dimensional lattice, and they allowed the simulated cells to both move and proliferate. Cell–to–cell adhesion was introduced by including a mechanism where the simulated cells could adhere to nearest neighbour simulated cells, effectively reducing their motility. Khain *et al.*[42,43] applied this model to investigate the behaviour of glioma cells in a two–dimensional scratch assay, predicting the location and speed of the leading edge of the expanding glioma cell colony. In another study, Simpson *et al.*[23] extended Khain’s model to investigate the migration of MCF–7 breast cancer cells in a three–dimensional Transwell^®;^ apparatus [23]. Although both these recent modelling studies incorporated a realistic cell–to–cell adhesion mechanism, there is no widely accepted protocol for designing experiments that allow us to independently quantify the contributions of cell motility, cell–to–cell adhesion and cell proliferation in expanding cell colonies [22,23,42-44]. We hypothesise that collecting and analysing several sets of experimental data describing the same experimental procedure may be required in order for us to independently quantify the role of these mechanisms.

In this work we use a circular barrier assay [45,46] to investigate the interplay between cell motility, cell–to–cell adhesion and cell proliferation mechanisms in expanding colonies of MM127 melanoma cells. We take a systematic approach that uses multiple types of data to identify each of these mechanisms separately by performing two sets of experiments. In our first set of experiments, we use Mitomycin–C to suppress cell proliferation so that we can separate the roles of cell motility and cell–to–cell adhesion from the role of cell proliferation. We attempt to quantify the roles of cell motility and cell–to–cell adhesion by extracting information about the location of the leading edge of the expanding colony and detailed cell density profiles throughout the entire cell colony. We find that these approaches do not provide sufficient information to identify the rate of cell motility and strength of cell–to–cell adhesion, and that additional data, including a measurement of the degree of cell–to–cell clustering, is required. Once we have obtained estimates of the cell motility rate and cell–to–cell adhesion strength we use a second set of experiments, in which proliferation is not suppressed, to estimate the rate of cell proliferation. Finally, given our independent estimates of the cell motility rate, strength of cell–to–cell adhesion and the cell proliferation rate, we confirm that our estimates allow us to accurately predict the observed spatial expansion in the experiments by comparing the predicted location of the leading edge and the predicted cell density profiles from our parameterised mathematical model to our experimental measurements.

## Results

### Identifying the mechanisms controlling the expansion of melanoma cell colonies

The spatial expansion of melanoma cell colonies is a complex process that is influenced by various mechanisms including cell motility, cell proliferation and cell–to–cell adhesion [47,48]. Although all three mechanisms are thought to play a critical role [47,48], it is unclear how the contributions of each of these three mechanisms can be identified and measured in a quantitative framework [7]. In this work, we use a combined experimental and mathematical modelling approach to distinguish between, and to quantify the role of, each mechanism.

**Figure 1:**
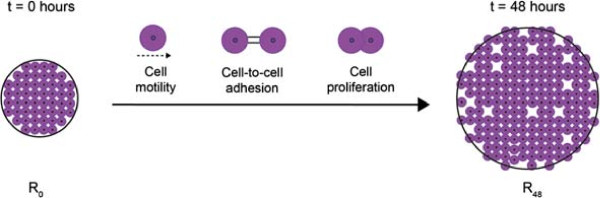
**Cell motility, cell–to–cell adhesion and cell proliferation mechanisms drive cell colony expansion.** Schematic representation of the circular barrier assay illustrating the mechanisms influencing the expansion of a two–dimensional cell colony. Cells are placed inside the barrier which is lifted at *t*=0 hours allowing the colony of cells to expand outwards until *t*=48 hours. The degree of expansion can be quantified by measuring and comparing the radius of the colony, *R*_0_ and *R*_48_.

**Figure 2:**
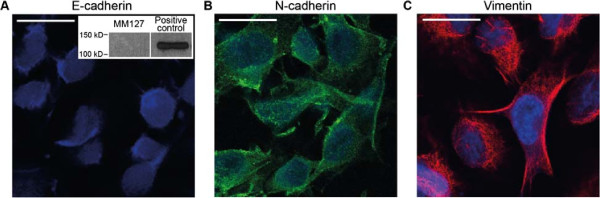
**MM127 melanoma cells express mesenchymal markers.** Immunofluorescence was used to examine expression of E–cadherin, a cell–to–cell adhesion protein uniquely expressed by epithelial cells **(A)**, N–cadherin, a cell–to–cell adhesion protein uniquely expressed by mesenchymal cells **(B)**, and vimentin, a protein that is uniquely expressed by mesenchymal cells **(C)** in MM127 cells. The scale bar corresponds to 25 *μ*m. MM127 melanoma cells were cultured in a circular barrier assay for *t*=48 hours on glass coverslips in 500 *μ*L cell medium. All sections were stained with DAPI (blue) to identify the cell nucleus. N–cadherin and vimentin expression are indicated by the green and red staining, respectively. Western blot was used to examine the expression of E–cadherin protein in MM127 cells (Inset in A).

To observe the spatial expansion of melanoma cell colonies, we performed several experiments using a circular barrier assay [45,46]. Figure 1 illustrates a schematic of the barrier assay. Human malignant melanoma cells (MM127, [49-51]) were placed inside the barrier at *t*=0 hours. The barrier was then lifted, allowing the cell colony to expand outwards. The spatial expansion of the colony was measured at *t*=24 and *t*=48 hours by calculating the radius, *R*, of the expanding circular colony. In our work, we placed either 20,000 or 30,000 cells inside the barrier initially. To confirm the presence of cell motility and cell–to–cell adhesion proteins in the cell colony, we used immunofluorescence to examine the expression of E–cadherin, N–cadherin and vimentin [3,47,52,53]. E–cadherin, a cell–to–cell adhesion protein that is uniquely expressed by epithelial cells [52], was not detected (Figure 2A). Western blot analysis (Figure 2A inset) confirmed the absence of E–cadherin [52]. In contrast, N–cadherin and vimentin, proteins that are uniquely expressed by mesenchymal cells, were detected (Figure 2B–C). The expression of N–cadherin suggests that cell–to–cell adhesion plays a role in this system, while the presence of vimentin is consistent with our initial assumption that the cells are motile [3,47,52,53]. In addition to the immunofluorescence results, we also visually identified that significant proliferation occurred during the barrier assays which we confirm during our later analysis (See section *Estimating the rate of proliferation*).

### Modelling the spatial expansion of a melanoma cell colony

**Figure 3:**
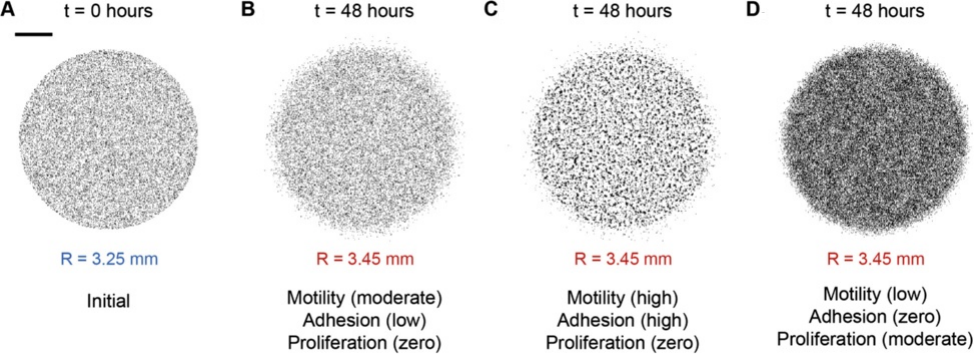
**Multiple combinations of *D*, *q* and *λ* result in the same extent of spatial expansion.** A circular barrier assay initialised with 20,000 cells was simulated using the mathematical model. The initial distribution of 20,000 simulated cells at *t*=0 hours is shown in **(A)**. The scale bar corresponds to 1.5 mm. Simulation snapshots shown in **(B–D)** illustrate three different combinations of the cell motility rate, cell–to–cell adhesion strength and cell proliferation rate used to replicate the experiments over *t*=48 hours. All three simulations result in a similar extent of spatial expansion from *R*=3.25 mm at *t*=0 hours to *R*=3.45 mm at *t*=48 hours. Simulations were performed with **(B)** moderate motility, low cell–to–cell adhesion strength and zero proliferation; *D*=405*μ*m^2^hour^−1^, *q*=0.1, *λ*=0hour^−1^, **(C)** high motility, high cell–to–cell adhesion strength and zero proliferation; *D*=810*μ*m^2^hour^−1^, *q*=0.8, *λ*=0hour^−1^, and **(D)** low motility, zero cell–to–cell adhesion and moderate proliferation; *D*=162*μ*m^2^hour^−1^, *q*=0, *λ*=0.035hour^−1^.

To interpret our experimental observations we used a discrete random walk model to simulate the expansion of the melanoma cell colonies [23,42,43,54]. The random walk model describes how a simulated cell can undergo specific events in a sequence of random steps [55]. These events include adhesive motility and proliferation, and we note that all of these mechanisms are simulated within a framework that incorporates realistic crowding effects [23,42,43,54]. We simulate these mechanisms on a two–dimensional square lattice with lattice spacing *Δ*. We estimate *Δ* by measuring the diameter of the cell nucleus using ImageJ [56], giving *Δ* = 18*μ*m [see Additional file 1]. Simulations of the experiments were performed on a lattice of size 867×867, whose dimensions correspond to the 15,600*μ*m diameter of well in a 24–well plate (15,600/18≈867). The simulations were initialised by placing either 20,000 or 30,000 simulated cells inside a circle located at the centre of the lattice. The radius of the initial circle was 3.25 mm, which corresponds to the average initial radius of the cell colony for both initial densities [see Additional file 1]. To reflect the way that the experiments were initiated, simulated cells were placed uniformly at random inside the circle so that the initial distribution of simulated cells matched the initial conditions in the experiments as accurately as possible. For example, if the initial radius of the circle is 3.25 mm, we represent this using a circle whose diameter corresponds to 180 lattice sites since 180≈325000/18. Hence, the total number of lattice sites inside that circle is *π*180^2^≈101736 and we randomly occupy 19.65% of these sites to mimic an experiment initialised with 20,000 cells since 19.65*%*=100×(20,000/101736). Similarly, we randomly occupy 29.49% of these sites to mimic an experiment initialised with 30,000 cells since 29.49*%*=100×(30,000/101736). Figure 3A illustrates the initial distribution for a simulation initialised with 20,000 simulated cells.

The model incorporates crowding effects by permitting each lattice site to be occupied by, at most, one simulated cell [21,43,57]. A random sequential update algorithm was used to perform the simulations [58] using the following algorithm. If there are *N*(*t*) simulated cells at time *t*, during the next time step of duration *τ*, *N*(*t*) simulated cells are selected at random, one at a time, and given the opportunity to move with probability *P*_*m*_(1−*q*)^*a*^. The random sequential update methods means that not all *N*(*t*) simulated cells are always selected during every time step; sometimes a particular simulated cell will be selected more than once per time step [58]. Here, 0≤*P*_*m*_≤1 is the probability that an isolated simulated cell moves a distance of one cell diameter, *Δ*, during a time interval of duration *τ*. The strength of adhesion is governed by the parameter 0≤*q*≤1, and 0≤*a*≤4 is the number of occupied nearest–neighbour lattice sites surrounding the simulated cell in question. When *q*=0, there is no cell–to–cell adhesion and nearest neighbour cells do not adhere to each other. As *q* increases, the strength of cell–to–cell adhesion increases, and nearest–neighbour cells adhere more tightly to each other. If the opportunity to move is successful and the target site is vacant, a simulated cell at position (*x*,*y*) steps to (*x*±*Δ*,*y*) or (*x*,*y*±*Δ*) with each target site chosen with equal probability of 1/4. Once the *N*(*t*) potential motility events have been assessed, another *N*(*t*) simulated cells are selected at random, one at a time, and given the opportunity to proliferate with probability 0≤*P*_*p*_≤1. If the opportunity to proliferate is successful, the proliferative simulated cell attempts to deposit a daughter simulated cell at (*x*±*Δ*,*y*) or (*x*,*y*±*Δ*) with each target site chosen with equal probability of 1/4.

In this work, we interpret the parameters describing cell motility and cell proliferation in our model using standard measures. The cell motility rate is quantified in terms of the cell diffusivity, *D*[17], which is related to the parameters in our model by *D*=(*P*_*m*_*Δ*^2^)/(4*τ*) [7,55]. Similarly, the rate of cell proliferation is given by *λ*=*P*_*p*_/*τ*[7,57]. Typical values of *D* are often reported to be of the order, *D*=1000*μ*m^2^hour^−1^[2,17]; however, particular estimates of *D* are known to vary by as much as to two orders of magnitude [1,2,7,57]. A typical doubling time, *t*_*d*_=log_e_(2)/*λ*, for melanoma cells is thought to be approximately 34 hours [59]. We note that while typical values of *D* and *λ* are sometimes reported in the literature, there are no such estimates of the strength of cell–to–cell adhesion, *q*[23].

In our analysis, we measure and quantify the dimensional cell density, *c*^∗^(*r*,*t*), where *r* describes the radial position (*μ*m) and *t* is time (hours). To measure the dimensional cell density, we consider a region of area *A*. In each region, we count the total number of cells, *N*, and divide through by the area to give *c*(*r*,*t*) = *N*/*A* cells *μ*m^−2^. In all cases, we convert the dimensional cell density into an equivalent non–dimensional cell density, *c*(*r*,*t*), by scaling with the carrying capacity density *K*. This gives *c*(*r*,*t*)=*c*^∗^(*r*,*t*)/*K*. We approximate the carrying capacity as the maximum packing density of circular–disk–like cells with diameter 18 *μ*m on a two–dimensional square lattice, giving *K*=1/*Δ*^2^=3×10^−3^ cells *μ*m^−2^[7]. In some regions where the cell density is approximately spatially uniform, we will refer to the non–dimensional cell density as a function of time only, *c*(*t*) [7]. This is particularly useful when we estimate the proliferation rate since we focus on regions in the middle of the colony where the spatial distribution of cells is relatively uniform so that locally we have *c*(*r*,*t*)≈*c*(*t*) [7].

Initially, we used the mathematical model to investigate whether a simple visual comparison of the simulated circular barrier assays for typical choices of *D*, *q* and *λ* could provide any insight into the factors affecting the spatial expansion of the experimental melanoma cell colony. Simulations in Figure 3B–D show three different realistic parameter combinations of *D*, *q* and *λ*. For these simulations we measure the extent of the spatial expansion of the colony by measuring the radius of the colony, *R*. Results in Figure 3B–D show that the spatial expansion after *t*=48 hours is equivalent for these different choices of *D*, *q* and *λ*. This observation suggests that there are multiple combinations of cell motility, cell–to–cell adhesion and cell proliferation parameters which could replicate the experiment results and therefore a simple visual inspection of the population is insufficient to identify the mechanisms influencing the expansion of the cell colony. To overcome this important limitation, we identified multiple types of data that could be extracted from the experiments. We will now describe each of these types of data and assess whether they are able to identify a unique set of *D*, *q* and *λ* parameters.

### Estimating the rate of cell motility and strength of cell–to–cell adhesion

To distinguish between the roles of cell motility and cell–to–cell adhesion, we considered experiments where cell proliferation was suppressed by performing the barrier assays with Mitomycin–C pretreated cells [7,60]. For each experiment we estimated the position of the leading edge of the expanding colony, the cell density profile along a transect throughout the entire expanding colony as well as measuring the degree of cell–to–cell clustering within the colony.

#### Data type 1: Location of the leading edge

**Figure 4:**
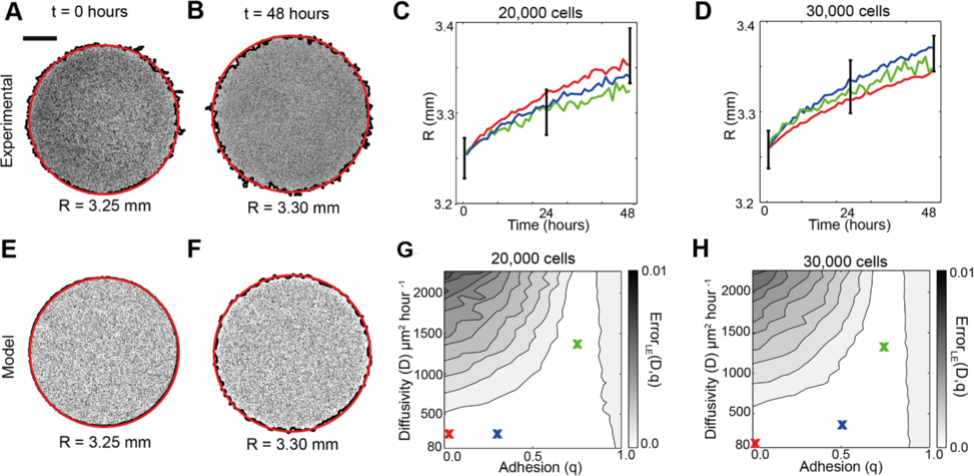
**The radius of the colony does not allow us to uniquely estimate *D* and *q*.** All results correspond to experiments where the cells were pretreated with Mitomycin–C to prevent cell proliferation. Images in **(A–B)**, show the entire cell colony for experiments initialised with 30,000 cells at *t*=0 and *t*=48 hours, respectively. The scale bar corresponds to 1.5 mm. An equivalent model simulation of the experiment is shown in **(E–F)**, using *D*=243*μ*m^2^hour^−1^, *q*=0 and *λ*=0hour^−1^. For all images in **(A–D)**, the detected location of the leading edge using the image analysis software is shown in black, while the red circle corresponds to the equivalent circle with the same area as enclosed by the leading edge. Results in **(C)** and **(D)** show the time evolution of the average radius of the expanding colony detected in the experiments. The error bars correspond to one standard deviation from the mean (black). Corresponding model simulations which match the experimental results are superimposed (colour lines). Simulation results were averaged over three identically–prepared realisations using three different combinations of parameters which are shown by the coloured crosses on the error surfaces in **(G)** and **(H)**. The model simulations in **(C–D)** were generated using *D*=162*μ*m^2^hour^−1^ and *q*=0 (red), *D*=162*μ*m^2^hour^−1^ and *q*=0.3 (blue) and *D*=1215*μ*m^2^hour^−1^ and *q*=0.8 (green), while solutions in **(D)**, were generated using *D*=81*μ*m^2^hour^−1^ and *q*=0 (red), *D*=243*μ*m^2^hour^−1^ and *q*=0.5 (blue), and *D*=1215*μ*m^2^hour^−1^ and *q*=0.8 (green), respectively. The error surfaces in **(G)** and **(H)** show Error_*LE*_(*D*,*q*), given by Equation 3, for various values of *D* and *q*. The error surfaces were by averaging the radius estimates from three experimental replicates and three identically–prepared model realisations.

The area enclosed by the leading edge of an expanding cell colony is a standard tool used to quantify the rate of cell colony expansion [7,61,62]. To determine the location of the leading edge we used image analysis software to analyse the experimental images showing the entire colony [see Additional file 1] [62]. Images in Figure 4A–B show the position of the leading edge detected at *t* = 0 and *t* = 48 hours, respectively. In both cases, the image analysis software accurately detects the position of the leading edge. For each experimental image we calculated the area enclosed by the detected leading edge, *A*, and converted the estimate of *A* into an estimate of the radius of the expanding colony, *R*, by assuming that the cell colony maintained a circular shape, giving $R=\sqrt{A/\pi }$, [7,62]. The estimates of the radius of the expanding colony are shown by the equivalent circular areas superimposed in Figure 4A–B. The excellent match between the detected leading edge and the corresponding equivalent circular area confirms that the cell colony maintains an approximately circular shape during the experiments. We repeated the leading edge detection procedure for all experimental images at *t*=0, 24 and 48 hours for both initial cell densities. Results in Figure 4C–D show how the estimates of *R* vary with time, indicating that the average radius of the expanding colony in the absence of proliferation increases gradually over *t*=48 hours. For the experiments initialised with 20,000 cells, the average radius increased from 3.25 mm to 3.30 mm, while the average radius of the expanding colony in the experiments initialised with 30,000 cells increased from 3.25 mm to 3.36 mm.

To investigate whether information about the radius of the expanding colony is sufficient to parameterise the cell diffusivity and strength of cell–to–cell adhesion, we used the mathematical model to perform several simulations to replicate the experiments where we varied the values of *D* and *q*. We initially considered a range of *D* values, approximately within the interval 0<*D*≤3000*μ*m^2^hour^−1^. We chose this range since typical reported values of the cell diffusivity are of the order 1000 *μ*m^2^hour^−1^[2,17] which means that our initial range of possible cell diffusivity values would encompass such typical values. To determine the appropriate values of *D*, we restricted our estimates so that each potential value of *D* corresponded to an integer number of simulation time steps, *b* = *T*/*τ*, where *T* = 48 hours is the total duration of the simulation. For example, *D* = 81*μ*m^2^hour^−1^ corresponds to a simulation where we chose *P*_*m*_ = 1 and *τ* = 1 hour, giving *b* = 48/1 = 48 simulation steps. Similarly, *D* = 810*μ*m^2^hour^−1^ corresponds to a simulation where we chose *P*_*m*_ = 1 and *τ* = 1/10 = 0.1 hour, giving *b* = 48/0.1 = 480 simulation steps. After some initial parameter investigations (not shown), we simulated the experiments by focussing on 30 equally–spaced values of *D* between 81 and 2430 *μ*m^2^hour^−1^. Since values of *q* are unknown, we choose to simulate the model using 11 equally spaced values of *q* between 0 and 1 to account for all possible values of the cell–to–cell adhesion strength.

For each different parameter combination, we simulated the experiments and averaged the results using three identically–prepared realisations of the model. Using the same image analysis procedure that was applied to the experimental images [7,62], we detected the leading edge of the simulated experiment, and calculated the area enclosed by the leading edge to determine *R*. Figure 4 E–F show two snapshots from a single realisation of the model with *D* = 243*μ*m^2^hour^−1^, *q* = 0 and *λ* = 0hour^−1^ at *t* = 0 and *t* = 48 hours. The equivalent circular area is also superimposed on the simulated colony. We observe again that the image analysis software is able to detect the position of the leading edge and that the equivalent radius estimate of the expanding colony is a good approximation of the location of the leading edge. In all cases we repeated our simulations for smaller values of *τ* while keeping the ratio of *P*_*m*_/*τ* constant. This exercise confirmed that our simulations were independent of the temporal discretisation.

To compare the simulation results with our experimental measurements, we assessed the goodness of fit between the experimental measurements and the model simulations using an estimate of the least–squares error, Error_*LE*_(*D*,*q*). This allowed us to determine whether there is an optimal choice of *D* and *q* in the model which matches the edge detection data [see methods *Assessing goodness of fit* and Equation 3]. In each case, the average radius of the expanding cell colonies, using three experimental replicates, was compared to the average radius of the expanding simulated cell colonies, again using three simulation replicates. Results in Figure 4G–H show the error surface, Error_*LE*_(*D*,*q*) for barrier assays initialised with 20,000 and 30,000 cells, respectively. We expect that any optimal choice of *D* and *q* would correspond to a unique minimum on the error surface. However, we observe that the low error region, for both initial cell densities, is very wide and there is no such unique minimum. For example, there is little distinction between simulations using combinations of *D* between 80 and 500 *μ*m^2^hour^−1^, and for *q* between 0 and 1, confirming that there is no unique choice of *D* and *q* to match our experimental data.

To illustrate this redundancy, we simulated the experiment using three different combinations of *D* and *q*. For example, to describe the experiments initialised with 20,000 cells, we performed simulations with *D* = 81, 162 and 810 *μ*m^2^hour^−1^ and *q* = 0, 0.3 and 0.8, respectively. In all cases, *λ* = 0 hour^−1^. The simulation results superimposed in Figure 4C–D show the average radius of the simulated expanding colonies compared to the experimental results. All three combinations of *D* and *q* match the experimental data, confirming that there are multiple combinations of *D* and *q* which accurately replicate the experimental data. In summary, these results illustrate that calibrating a mathematical model using the position of the leading edge alone is inadequate to uniquely identify the rate of cell motility and strength of cell–to–cell adhesion [62].

#### Data type 2: Cell density profiles

**Figure 5:**
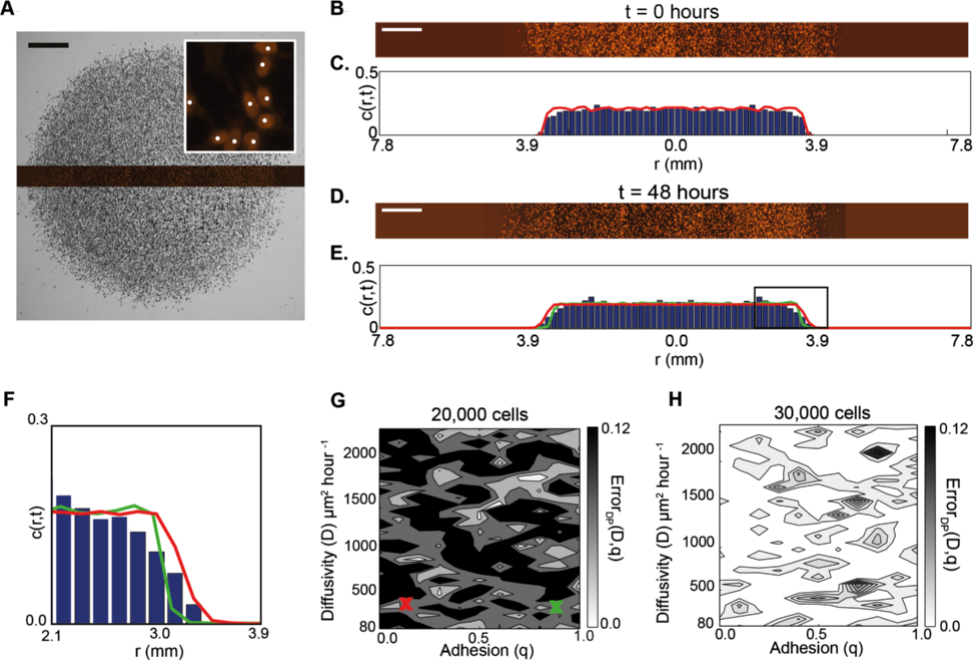
**Cell density profile data does not allow us to uniquely estimate *D* and *q*.** All results correspond to experiments where cells were pretreated with Mitomycin–C to prevent cell proliferation. Cell density profiles were extracted from each experiment by partitioning the transect into 98 rectangular regions. The transect is the dark region shown in **(A)** passing through the entire cell colony. The scale bar corresponds to 1.5 mm. The magnified image inset in **(A)** shows several cells which have been identified by image analysis software (white dots). Experimental transects at *t*=0 and *t*=48 hours are shown in **(B)** and **(D)** for experiments initialised with 20,000 cells. The scale bar corresponds to 1.5 mm. Histograms showing the experimental cell density profile along the transect are shown in **(C)** and **(E)**. Each experimental cell density profile was averaged using three experiments as described in the text. The corresponding model predictions are superimposed at *t*=0 hours in **(C)** and at *t*=48 hours in **(E)** using five identically–prepared realisations of the model. Both curves correspond to simulations where *D*=243*μ*m^2^hour^−1^. The red curve in **(E)** corresponds to weak cell–to–cell adhesion (*q*=0.1) and the green curve corresponds to strong cell–to–cell adhesion (*q*=0.9). The leading edge in **(E)** is magnified in **(F)**. The error surfaces in **(G)** and **(H)** show Error_*DP*_(*D*,*q*), given by Equation 4, for various values of *D* and *q*. Simulation results are averaged over five identically–prepared realisations. The red and green crosses in **(G)** correspond to the two model solutions superimposed in **(E)**.

An alternative approach to estimate the model parameters, which does not solely rely on the location of the leading edge of the expanding cell colony, is to extract detailed information about the location of individual cells throughout the population and to construct a cell density profile throughout the entire expanding colony [7,18]. This allows us to compare additional information about the distributions of cells in the experiments. For all experiments, a high magnification image of a transect across the center of the colony stained with Propidium Iodide was acquired, as illustrated in Figure 5A. This allowed us to identify the location of individual cells within the expanding colony [7]. Each transect was partitioned into 98 sections along the transect axis, where each section had length 160 *μ*m and width 260 *μ*m. Figure 5A shows a schematic representation of the transect through the centre of the colony. Image analysis was used to count the number of cells in each section of the transect which allowed us to calculate the dimensional cell density, *c*^∗^(*r*,*t*), and the corresponding non–dimensional cell density profile, *c*(*r*,*t*), [see section *Modelling the spatial expansion of a melanoma cell colony* and Additional file 1] [7].

To determine an averaged cell density profile for each experiment, we extracted three cell density profiles from three experimental replicates [see Additional file 1]. Each density profile was divided at the centre of the profile so that each half profile described one–half of the entire cell density profile from the centre of the colony (*r*=0) to the leading edge (*r*=*R*). The corresponding non-dimensional cell density profiles from all six half profiles were averaged. Figure 5B–C shows an experimental transect image at *t*=0 hours and the corresponding averaged cell density profile using three replicates. For both initial cell densities, we observe that the density distribution at *t*=0 hours is approximately uniform throughout the colony which is consistent with our experimental procedure where we attempted to place the cells inside the barrier as evenly as possible. The experimental transect image and corresponding averaged cell density profile at *t*=48 hours are shown in Figure 5D–E. Here, we see that the leading edge of the cell colony has expanded as observed previously in the leading edge analysis (Figure 4C–D).

Simulated cell density profiles were extracted from the mathematical model using the same process applied to the experimental transects. Simulations were performed using the same combinations of *D* and *q* as for the analysis of the leading edge data (Figure 4G–H). The averaged experimental density profile at *t*=0 hours for each initial cell density was used to initiate the model simulations. One realisation of the simulated density profile is superimposed onto the averaged experimental histogram in Figure 5C. In all cases, the simulated cell density profiles for each parameter combination were averaged over five identically–prepared realisations of the model. Two averaged simulated cell density profiles for simulations with *D* = 81 *μ*m^2^hour^−1^ at *t*=48 hours using low cell–to–cell adhesion strength, *q*=0.1, and strong cell–to–cell adhesion strength, *q*=0.9, are superimposed onto the corresponding experimental cell density profile in Figure 5E. A visual comparison of the experimental density profile and the two simulation profiles provides no definite indication of whether a low value of *q* or high value of *q* best matches the experimental measurement. This observation is confirmed by examining the magnified image of the leading edge in Figure 5F where we again see that it is not obvious whether the low *q* or the high *q* matches the measurements. These results indicate that comparing density profile alone information may not be able to distinguish an optimal parameter combination.

To compare the experimental and simulation density profiles for a broader combination of parameters we used an estimate of the least–squares error, Error_*DP*_(*D*,*q*), to determine whether there is an optimal choice of *D* and *q* to match the cell density data [see methods*Assessing goodness of fit*and Equation 4]. For each combination of *D* and *q*, we calculated Error_*DP*_(*D*,*q*) and compared the averaged simulated cell density profile with the corresponding averaged experimental profile to produce the error surfaces in Figure 5G–H. The error surfaces confirm that there is no well–defined error, indicating that there is no optimal choice of *D* and *q* which indicates that density profiles cannot be used alone to estimate *D* and *q*.

#### Data type 3: Degree of cell clustering

The degree of cell–to–cell clustering within a group of cells is thought to indicate the presence of cell–to–cell adhesion [43,63]. However, we note that there is no standard, widely accepted measure of cell clustering that has been proposed for this purpose when dealing with experimental data [23,43,63,64].

**Figure 6:**
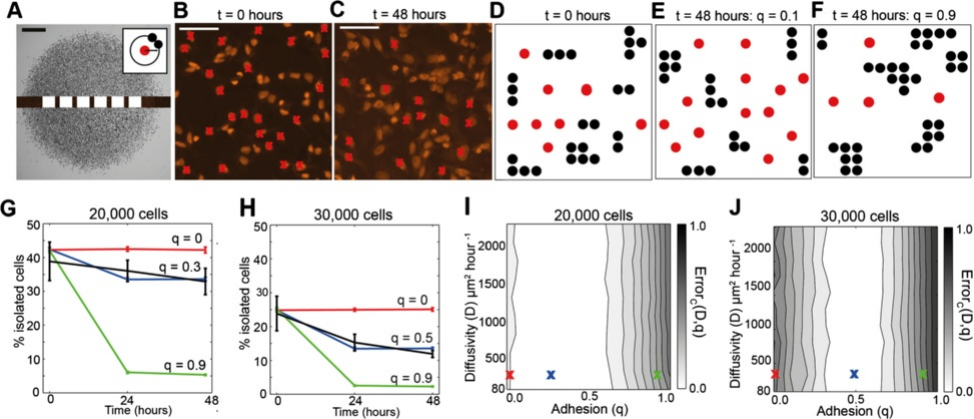
**The degree of cell clustering allows us to estimate *q*.** All results correspond to experiments where cells were pretreated with Mitomycin–C to prevent cell proliferation. Isolated cells were identified in several regions along the experimental transects, as shown to scale in **(A)**. The scale bar corresponds to 1.5 mm. The inset in **(A)** illustrates a schematic representation of our definition of an isolated cell that occupies a circular region of at least radius 18 *μ*m that contains no other cells. The inset in **(A)** is not to scale. Experimental snapshots in **(B–C)** show Propidium Iodide images at *t*=0 and *t*=48 hours, respectively, for experiments initialised with 30,000 cells. The scale bar corresponds to 0.1 mm. Red crosses indicate cells which were identified as isolated cells by the image analysis software. Snapshots of the model simulations are shown in **(D–F)**, at *t*=0 hours, at *t*=48 hours with weak cell–to–cell adhesion, *q*=0.1 and at *t*=48 hours with strong cell–to–cell adhesion, *q*=0.9. Simulations were performed using *D*=243*μ*m^2^hour^−1^. Red circles correspond to isolated cells, while black circles indicate all other connected cells. Results in **(G–H)**, show the time evolution of the average percentage of isolated cells for experiments initialised with 20,000 and 30,000 cells, respectively. The error bars correspond to one standard deviation from the mean (experimental – black, model – red, blue and green) and all simulation results were averaged over twenty realisations. Equivalent simulations of the mathematical model with no cell–to–cell adhesion (red lines) and strong cell–to–cell adhesion are superimposed (green lines). The best match solutions using *q*=0.3 and *q*=0.5, respectively, are shown in blue. The error surfaces in **(I)** and **(J)** show Error_*C*_(*D*,*q*), given by Equation 5, for various values of *D* and *q*. Simulation results are averaged over twenty identically–prepared realisations. The red, green and blue crosses in **(I)** and **(J)** correspond to the solutions superimposed in **(G)** and **(H)**, respectively.

In this work we propose to measure the degree of cell clustering by identifying isolated cells within the colony. We define an isolated cell to be a cell that appears to lack physical contact with other cells. For each experiment with Mitomycin–C pretreatment, we used image processing software to analyse six regions, containing cells stained with Propidium Iodide, located approximately in the centre of the colony. Each region has a size of 500 *μ*m× 2000 *μ*m. The approximate locations of the six regions are illustrated in Figure 6A. We note that in Figure 6 we adopt the convention that red circles correspond to isolated cells while black circles correspond to cells which appear to be connected to other cells within the colony. To determine the proportion of isolated cells in each region, we used image analysis software to count the total number of cells using the same procedure described in the cell density profile analysis. The number of isolated cells was counted using the image analysis software to detect those cells that occupied a circular region, of radius 18 *μ*m, that was not occupied any other cells. The inset in Figure 6A shows an isolated red cell which occupies a circular region, which has a radius of 18 *μ*m, that contains no other cells [see Additional file 1]. In all cases, we manually checked the image analysis results to ensure that all isolated cells were identified correctly. Snapshots from the region analysed, shown in Figure 6B–C at *t*=0 and *t*=48 hours, illustrate that the proportion of isolated cells decreases with time which suggests that these cells are more likely to form cell–to–cell contacts as the experiment proceeds. This observation is consistent with the idea that cell–to–cell adhesion plays an important role in the expansion of the MM127 colony. Results comparing the average percentage of isolated cells in the cell colony in each of the six regions are illustrated in Figure 6G–H, confirming that the proportion of isolated cells in the colony decreases over time.

To determine whether there is an optimal choice of *D* and *q* that matches our experimental measurements, we simulated the experiments using 11 equally–spaced values of *D* between 81 and 2430 *μ*m^2^hour^−1^, and 11 equally–spaced values of *q* between 0 and 1. All simulations were performed with *λ*=0hour^−1^ since we are dealing with Mitomycin–C pretreated cells. Image analysis software was used to automatically identify isolated cells in the simulations using exactly the same approach applied to the experimental images. Snapshots from the region analysed in each simulation are shown in Figure 6D at *t*=0 hours and in Figure 6E–F at *t*=48 hours using a low and high value of *q*, respectively. We observe that for a low value of *q*, the proportion of isolated simulated cells does not decrease with time. However, for a high value of *q* the proportion of isolated simulated cells decreases considerably. We repeated the simulations, averaging our results over twenty identically prepared realisations, for each different value of *q*, to determine an average estimate of the proportion of isolated simulated cells in the simulated cell colony at each time point.

The average percentage of isolated cells for three different values of *q* and *D*=243*μ*m^2^hour^−1^ are superimposed onto the experimental results in Figure 6G–H. For both initial densities, we observe that the simulation results with *q*=0 do not match our experimental results. Similarly, results with very high cell–to–cell adhesion strength, *q*=0.9, also do not match. To determine the optimal value of *q*, we used an estimate of the least–squares error, Error_*C*_(*D*,*q*), for each combination of *D* and *q* [see methods *Assessing goodness of fit*, Equation 5 and Additional File 1]. The error surfaces for each initial density are shown in Figure 6I–J. In contrast to our previous error surfaces for the leading edge and cell density profile analysis (Figure 4G–H; Figure 5G–H), our results show that there is a well defined value of *q* corresponding to a minimum in Error_*C*_(*D*,*q*) for both initial cell densities. This suggests that there is an optimal choice of *q* to match our observations. We also observe that our results are insensitive to the choice of *D* since the error surfaces in Figure 6I–J appear to be independent of the value of *D*. The error surfaces indicate that for the experiments initialised with 20,000 cells, the optimal choice of *q* is between *q*=0.1 and *q*=0.6 and for experiments initialised with 30,000 cells the optimal range is between *q*=0.3 and *q*=0.6. Simulation results using values of *q* in the middle of these ranges, *q*=0.3 and *q*=0.5, for experiments initialised with 20,000 and 30,000 cells, respectively, are superimposed in Figure 6G–H. The correspondence between the experimental measurements and the simulation data suggests that a low–to–moderate cell–to–cell adhesion strength is necessary to describe our measurements in the MM127 melanoma cell colony. Now that we have obtained an estimate of *q*, we can use this information to determine the associated range of *D* values using our results from the leading edge analysis (Figure 4G–H) which we will discuss in section *Predicting the spatial expansion of a melanoma cell colony*.

### Estimating the rate of proliferation

#### Data type 4: Cell density counts

**Figure 7:**
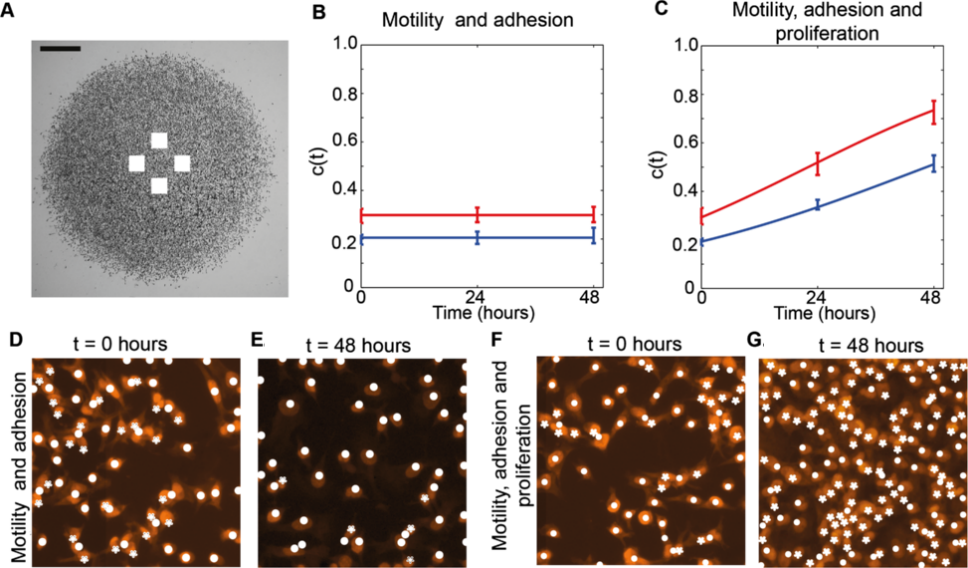
**Cell density measurements where cell proliferation is not suppressed allows us to estimate *λ*.** The approximate location of the subregions used to measure the cell density are shown in **(A)**, where the scale bar corresponds to 1.5 mm. Images in **(D–E)** show two subregions of dimensions 230 *μ*m× 230 *μ*m for experiments at *t*=0 hours and *t*=48 hours, where 30,000 cells, pretreated with Mitomycin–C, were initially placed inside the barrier. Equivalent images without Mitomycin–C pretreatment are shown in **(F–G)**. The Propidium Iodide staining is highlighted in orange. For each subregion, the number of cells was counted; white circles correspond to the cells automatically detected by the image analysis software and white stars indicate cells that were manually counted. The corresponding time evolution of the mean scaled density, *c*(*t*), is shown in **(B)** and **(C)**, where the error bars indicating one standard deviation from the mean. Blue and red data points correspond to the experiments initialised with 20,000 and 30,000 cells, respectively. Our analysis shows that the proliferation rate (*λ*) and the doubling time (*t*_*d*_=log_*e*_2/*λ*) for the experiments initialised with 20,000 cells is *λ*=0.0305hour^−1^ and *t*_*d*_=22.7 hours, and for experiments initialised with 30,000 cells is *λ*=0.0398hour^−1^ and *t*_*d*_=17.42 hours. The red and blue curves in **(B)** and **(C)** show the corresponding solution of the logistic equation, given by Equation 2, respectively.

To quantify the cell proliferation rate we considered experiments performed without Mitomycin–C pretreatment so that cell proliferation is not suppressed. Propidium Iodide stained transect images were used to identify individual cells located approximately at the centre of the colony. For each replicate experiment, the number of cells in four different subregions, each of dimension 230 *μ*m× 230 *μ*m, was counted and converted into a measurement of the non–dimensional cell density, *c*(*t*), [see Additional file 1]. Here, we report values of *c*(*t*), instead of *c*(*r*,*t*), since we are focusing on the centre of each colony away from the leading edge where the cell density is approximately spatially uniform [See section *Modelling the spatial expansion of a melanoma cell colony*] [7]. The approximate location and size of each subregion is illustrated in Figure 7A.

Images in Figure 7D–E show snapshots of cells pretreated with Mitomycin–C indicating that the number of cells does not increase or decrease over time. This confirms that Mitomycin–C pretreatment prevents proliferation and, importantly, did not cause cell death. Snapshots in Figure 7F–G, where the cells are not pretreated with Mitomycin–C, indicates that the number of cells increases dramatically over time. These visual observations are confirmed by examining the evolution of the non–dimensional cell density, *c*(*t*), in Figure 7B–C, where we again see that the cell density does not increase or decrease in cell colonies with no proliferation, and increases substantially in cell colonies with proliferation.

To estimate the proliferation rate, we note that the proliferation mechanism in our mathematical model is related to the logistic equation [57] and is given by

(1) $$\frac{dc(t)}{dt}=\mathrm{\lambda c}(t)(1-c(t)),$$

which has the solution

(2) $$c(t)=\frac{c(0)\exp(\mathrm{\lambda t})}{1+c(0)(\exp(\mathrm{\lambda t})-1)},$$

where *c*(0) is the initial non–dimensional cell density. To estimate the cell proliferation rate, we found the value of *λ* that minimised an estimate of the least–squares error between our experiments and the solution of the logistic equation [see methods *Assessing goodness of fit*, Equation 6 and Additional File 1]. For the experiments initialised with 20,000 cells without Mitomycin–C pretreatment, we found that *λ*=0.0305 (0.0278,0.0329)hour^−1^, here the uncertainty in our estimate is given as a range in parenthesis [7]. For the equivalent experiment with Mitomycin–C pretreatment we have *λ*=0.0002 (0,0.0015)hour^−1^. For the experiments initialised with 30,000 cells, we found *λ*=0.0398 (0.0338,0.0444)hour^−1^ for the experiments without Mitomycin–C pretreatment. For the experiments initialised with 30,000 cells, we found *λ*=0.0001 (0,0.0027)hour^−1^ for the experiments with Mitomycin–C pretreatment. The relevant logistic growth curves, given by Equation 2, are superimposed in Figure 7B–C and illustrate that the proliferation rate estimates obtained describe the observed changes in the experimental cell density over time. We note that our estimates of *λ* is associated with a doubling time, *t*_*d*_=log_*e*_2/*λ*, of 22.7 and 17.42 hours for experiments initialised with 20,000 and 30,000 cells, respectively.

### Predicting the spatial expansion of a melanoma cell colony

**Figure 8:**
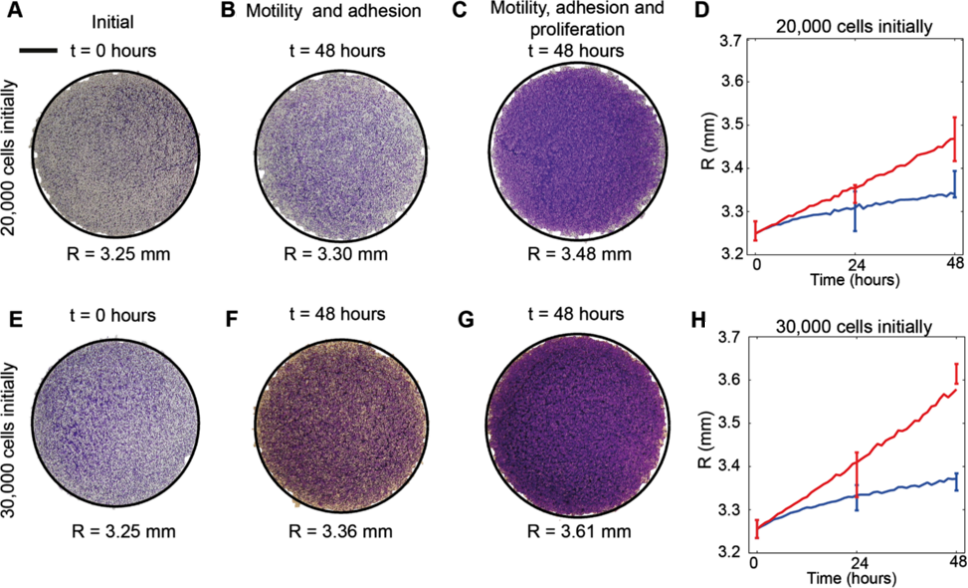
**Independent estimates of *D*, *q* and *λ* predict the spatial extent of the expanding colony.** Experimental measurements of the radius of the expanding colony were compared to predictions from the parameterised mathematical model using the parameter estimates determined previously. Results in **(A–C)** and **(E–G)** compare the position of the leading edge for experiments where 20,000 and 30,000 cells were initially placed inside the barrier, respectively. The scale bar corresponds to 1.5 mm. Images are shown at *t*=0 hours **(A,E)**, at *t*=48 hours for the experiments with Mitomycin–C pretreatment **(B,F)** and at *t*=48 hours without Mitomycin–C pretreatment **(C,G)**. The solid curves superimposed (black) on each image correspond to the relevant simulation which has been been converted into an equivalent circular area. In all cases, simulation results were averaged over three realisations. Results in **(D)** and **(H)** show the mean radius calculated from the experimental images at *t*=0, 24 and 48 hours, with error bars indicating one standard deviation from the mean. The corresponding average radius of the simulated expanding colony is superimposed in **(D)** and **(H)**. Blue lines correspond to experiments where proliferation was suppressed using Mitomycin–C pretreatment, while red lines correspond to experiments where proliferation was not suppressed. Simulations were averaged over three identically–prepared realisations. Simulations of the experiments initialised with 20,000 cells correspond to *D*=162*μ*m^2^hour^−1^, *q*=0.3 and *λ*=0.0305hour^−1^, and simulation of the experiments initialised with 30,000 cells correspond to *D*=243*μ*m^2^hour^−1^, *q*=0.5 and *λ*=0.0398hour^−1^.

We now test whether our independently–derived estimates of *D*, *q* and *λ* accurately predict the location of the leading edge and the cell density profiles of the expanding melanoma cell colony. Experimental images of the entire cell colony in Figure 8A–C, E–G compare the distribution of cells at *t*=0 and *t*=48 hours, both with and without Mitomycin–C pretreatment. We visually observe that the colonies without proliferation do not appear to expand as fast as the colonies with proliferation. The overall increase in the average radius of the expanding colonies without proliferation after *t*=48 hours is 2.2%. In contrast, the average radius increase in expanding melanoma cell colonies with proliferation is 9.1%. These results illustrate that proliferation plays a major role in the spatial expansion of melanoma cell colonies.

To compare our model predictions with the experimental measurements we combined our results using all the information obtained from different types of experimental data. For experiments initialised with 20,000 cells we estimated that *q*=0.3. We obtained an estimate of *D* from the error surfaces associated with the leading edge data Figure 4G. For *q*=0.3, the associated range of *D* which have the lowest error are between *D*=81 and *D*=567*μ*m^2^hour^−1^. Similarly, for experiments initialised with 30,000 cells, we estimated that *q*=0.5, giving a corresponding range of *D* values between *D*=81 and *D*=729*μ*m^2^hour^−1^. For both initial densities, we simulate the experiments with a value of *D* within the range obtained that gave the minimum error in Figure 4G,H. In summary, for experiments initialised with 20,000 cells, we estimate *D*=162*μ*m^2^hour^−1^, *q*=0.3 and *λ*=0.0305hour^−1^ and for experiments initialised with 30,000 cells, we estimate *D*=243*μ*m^2^hour^−1^, *q*=0.5 and *λ*=0.0398hour^−1^. We note that our estimates indicate some weak dependence on the initial numbers of cells since the values of the cell diffusivity, strength of cell–to–cell adhesion and proliferation rate all increase slightly as the initial numbers of cells placed inside the barrier increases.

We performed simulations of experiments using our estimates of *D*, *q* and *λ* to examine whether the parameterised mathematical model predicts the differences observed in the experiments where cell proliferation is suppressed compared with the observations when cell proliferation is allowed [see Additional file 1]. The predictions of the model, in terms of the average circular area enclosing the leading edge of the expanding colony, are superimposed onto the corresponding experimental image in Figure 8A–C, E–G showing that the parameterised model accurately matches the experimental observations. Analysing all images at *t*=0, 24 and 48 hours, we produced the data in Figure 8D,H comparing the radius of the expanding colony measured in the experiments with the predictions of the model. We note that the prediction of the mathematical model at *t*=48 hours for the experiments without Mitomycin–C pretreatment, initialised with 30,000 cells, slightly underestimated the experimental data. Despite this, overall our comparison indicates that the parameterised model predicts the time evolution of the radius of the expanding melanoma cell colony and captures the differences in our experiments where proliferation was either allowed or suppressed.

**Figure 9:**
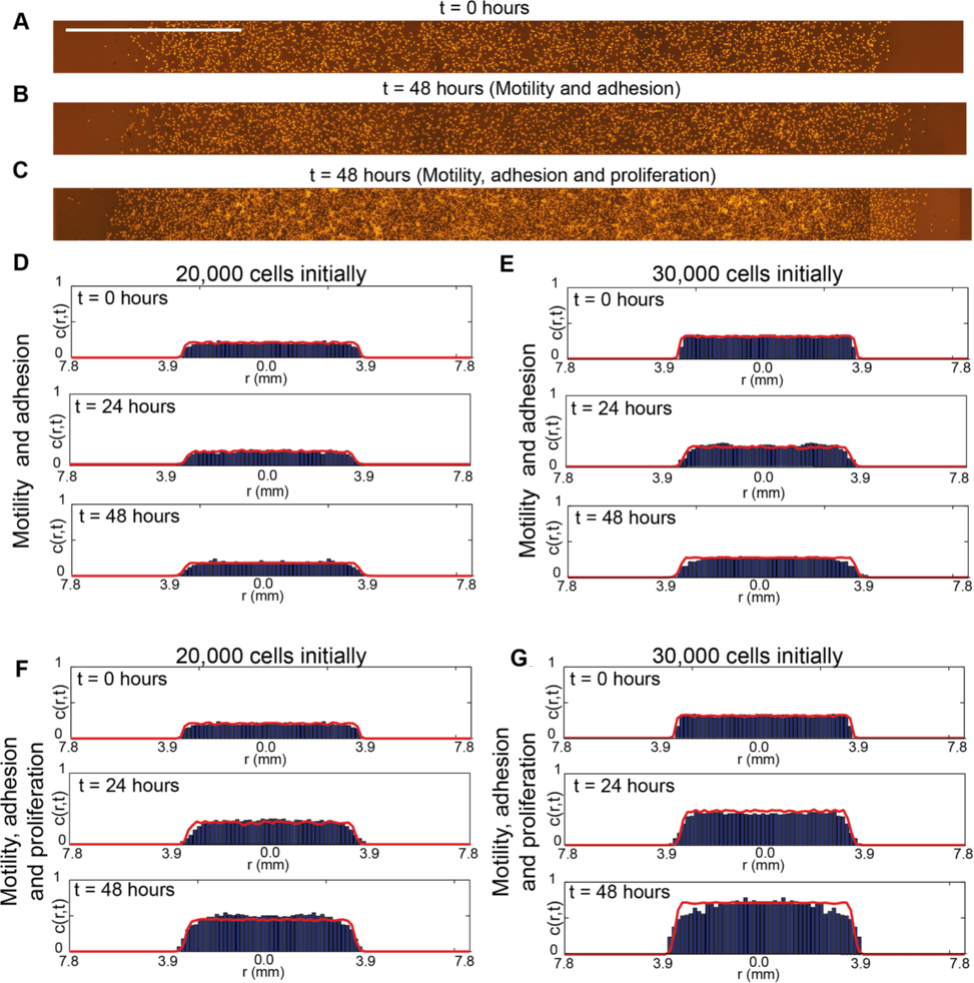
**Independent estimates of *D*, *q* and *λ* predict the density profiles in the cell colony.** Experimental measurements of the cell density profile are compared with the predictions of the mathematical model using the parameter estimates reported previously. Images of the transects for the experiments initialised with 30,000 cells are shown at *t*=0 hours **(A)**, at *t*=48 hours where proliferation was suppressed **(B)**, and at *t*=48 hours where proliferation was not suppressed **(C)**. The scale bar corresponds to 1.5 mm. Experimental histograms and the corresponding simulated cell density profiles for all sets of experiments at *t*=0, *t*=24 and *t*=48 hours, both with and without proliferation, are shown in **(D–G)**. Simulations were averaged over five identically–prepared realisations. Model simulations of the experiments initialised with 20,000 cells correspond to *D*=162*μ*m^2^hour^−1^, *q*=0.3 and *λ*=0.0305hour^−1^, and for experiments initialised with 30,000 cells, simulations correspond to *D*=243*μ*m^2^hour^−1^, *q*=0.5 and *λ*=0.0398hour^−1^.

We now test whether our parameterised model can predict the cell density profile throughout the entire expanding colony for all cases considered in our experimental program. Experimental images in Figure 9A–C highlight major visual differences between the distribution of cells in the experiments where we suppressed cell proliferation relative to the equivalent experiment where we allowed cell proliferation. The corresponding cell density profiles extracted from the experimental images are shown in Figure 9D–G. Equivalent simulations of these experimental conditions using our parameterised mathematical model are superimposed onto the experimental density profiles and we note that in all cases the match between the model prediction and the experimental measurements are excellent. This confirms that our parameterised mathematical model accurately predicts both the spatial extent of the expanding cell population and the distribution of individual cells within the expanding melanoma cell colony. Moreover, our approach can predict how differences in individual cell behaviour, such as the cell proliferation rate, affect the emergent properties of the expanding colony.

## Discussion

Despite compelling evidence that cell–to–cell adhesion plays an important role in many expanding cell populations, standard mathematical modelling approaches often neglect to include any such mechanism [1,2,7,16-18]. This may explain why reported estimates of the cell diffusivity can vary widely since these estimates have often been obtained by calibrating mathematical models which neglect to incorporate cell–to–cell adhesion [1,2,7,57]. To overcome these issues combined experimental and modelling approaches that can separately identify and quantify the roles of cell motility, cell–to–cell adhesion and cell proliferation are required.

In our work, we used a combined experimental and modelling approach to independently quantify the roles of cell motility, cell–to–cell adhesion and cell proliferation in an expanding colony of MM127 melanoma cells. Our experimental approach used a circular barrier assay, while our modelling approach incorporated cell–to–cell adhesion as well as cell motility and cell proliferation mechanisms. In contrast to previous approaches, we extracted multiple types of data from the same barrier assay and used these different kinds of data to attempt to independently quantify the cell motility rate, *D*, cell–to–cell adhesion strength, *q*, and proliferation rate, *λ*. To separate the role of cell proliferation from the roles of cell motility and cell–to–cell adhesion, we first performed a set of experiments where we suppressed proliferation to quantify *D* and *q*. We then performed a second set of experiments with proliferation to quantify the cell proliferation rate, *λ*. All experiments were repeated at two initial cell densities and each experiment was replicated three times.

We extracted three different types of data from experiments where proliferation was suppressed to identify *D* and *q*. Our first type of data was to estimate the area enclosed by the leading edge of the expanding colony to determine whether there was a unique choice of *D* and *q* that matched the experimental measurements. Our analysis of the leading edge data indicates that this commonly–used measurement is insufficient to uniquely identify *D* and *q* suggesting that additional data is required. It is important to recognise the limitations of the leading edge data since this is one of the most commonly–reported types of data [62]. In an attempt to overcome the limitations of the leading edge data, we extracted detailed cell density profiles throughout the entire colony. Our attempts to calibrate the mathematical model to these more detailed measurements also failed to identify a unique choice of *D* and *q* to match the measurements.

In an attempt to estimate the strength of cell–to–cell adhesion we then measured the degree of cell clustering in the expanding melanoma cell colony by measuring the proportion of isolated cells within the colony. We found this to be a convenient measure of the degree of cell clustering since isolated cells were easily identifiable using an automated image processing software. Our results indicated that a low to moderate cell–to–cell adhesion strength in the mathematical model provided the best match to the measurements. Once we had estimated *q* we were then able to identify a range of *D* from combining our results regarding the degree of cell clustering with our results describing the time evolution of the position of the leading edge of the expanding cell colony.

To estimate the proliferation rate we measured the temporal change in cell density in a set of experiments where cell proliferation was not suppressed. Our estimates of *λ* indicate that the role of cell proliferation in the experiments is considerable since the doubling time is approximately 20 hours and experiments were performed over a period of 48 hours. We used our estimates of *D*, *q* and *λ* to make predictions about the expansion of the melanoma cell colony which confirmed that our parameterised mathematical model matched the experiments and was able to accurately predict differences between the results when cell proliferation was suppressed compared to experiments when cell proliferation was allowed. In summary, we showed that the spatial expansion of the melanoma cell colony is significantly enhanced by cell proliferation. We also found that our estimates of *D*, *q* and *λ* are weakly dependent on the initial cell density in the experiments. This is an important observation since many experimental and modelling studies do not consider the affect of the initial density in a barrier assay; however, our results illustrate that these effects could be important [7].

One of the advantages of our mathematical modelling approach are that the discrete model explicitly represents cell motility, cell–to–cell adhesion and cell proliferation processes. The model is straightforward to implement and provides us with a relatively straightforward physical interpretation of how different mechanisms acting at the level of an individual cell contributes to the population-level expansion of the cell colony. Most importantly, when combined with appropriate experimental data, our model allows us to separately identify, and quantify, the role of each individual cell–level mechanism in the expanding cell colony.

**Figure 10:**
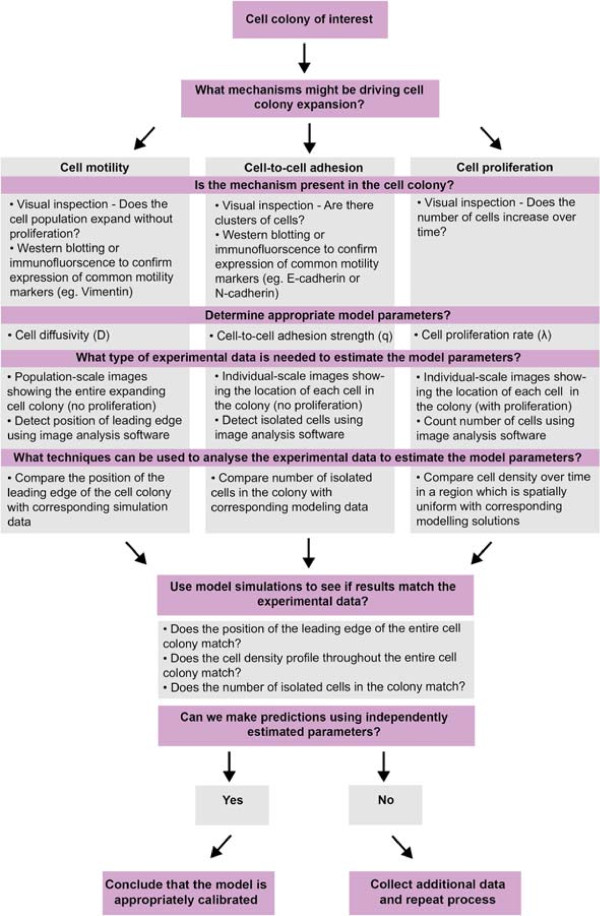
**Framework illustrating a systematic approach that can be used to independently identify and quantify the mechanisms driving cell colony expansion.** The mechanisms thought to be driving the expansion of a selected cell colony are identified and confirmed using visual inspection or other advanced experimental techniques. A mathematical modelling approach is selected and appropriate model parameters defined. Various experimental data is extracted to estimate the model parameters. For each additional mechanism considered, we anticipate that we will require at least one further piece of information from the experiments to quantify the role of that particular mechanism. The experimental data is analysed by extracting simulation data from the mathematical model and testing how well the model predictions match with the experimental data. If the modelling predictions provide a good match to the experimental data we stop the process. Alternatively, if the modelling predictions do not uniquely match the experimental data, we repeat the process iteratively using additional types of data extracted.

A schematic illustration of our systematic approach for identifying and quantifying the roles of cell motility, cell proliferation and cell–to–cell adhesion is given in Figure 10. Our approach can be summarised in the following way: for a particular cell colony we begin with a hypothesis about which particular mechanisms might be involved in the expansion of that colony. We then attempt to determine whether these putative mechanisms are present in the cell colony using visual inspection of the experimental data or immunofluorescence techniques. If the mechanisms are present, we identify an appropriate modelling method and include model parameters which control that particular mechanism of interest. Next, we attempt to determine what type of experimental data could be used to estimate the relevant model parameters. After we have extracted this data, we use the mathematical model to simulate the experiment in an attempt to understand if a particular choice of parameter(s) can predict the observed behaviour. If the model predictions give a good agreement with the experimental observations we stop the process. Otherwise, if we find that we do not enough types of data to completely parameterise the model we should collect more types of different data and repeat the process iteratively. If this approach fails, then the experimental or modelling approach should be reconsidered. In our case, we found that using this approach implied that we had to consider multiple data types to independently identify and quantify the mechanisms controlling the expansion of a melanoma cell colony. We suggest that this general framework could be used to analyse other biological processes.

A clear consequence of our work is that while it is relatively simple to incorporate detailed mechanisms into a mathematical model, it becomes increasingly difficult to independently identify the contributions of each mechanism in the mathematical model using experimental data. However, we anticipate that for every additional mechanism and parameter incorporated into a mathematical model of collective cell behaviour, further additional experimental data types are required so that we can parameterise the mathematical model. This quickly becomes infeasible when multiple mechanisms are considered. For example, in our work, we incorporated three different factors into the mathematical model (cell motility, cell-to–cell adhesion and cell proliferation) and we found that we needed to consider at least four different data types to quantify these mechanisms.

Our work has been aimed at improving our understanding of how 2D *in vitro* colonies of melanoma cells expand. An important limitation of our work is that it cannot be directly applied to three–dimensional (3D) *in vitro* experiments since the techniques used to quantify the cell motility rate, strength of cell–to–cell adhesion and cell proliferation rate in 2D cell colonies do not directly translate to 3D cell colonies. For example, the leading edge of a cell colony in 2D is straightforward to locate using standard imaging software and analysis [62]. In 3D, however, detection of the edge, or surface, of the cell colony is significantly more challenging and requires more sophisticated imaging software and more detailed image analysis algorithms [65-68]. Similar difficulties are also relevant in terms of locating and counting individual cells within a 3D colony [65,69].

Our work has been focused on interpreting *in vitro* measurements of cell colony expansion [65]. Extending our approach to *in vivo* colony expansion would involve dealing with many more mechanisms that are not present in the *in vitro* system [65,69]. These additional mechanisms could include more complicated signalling pathways that regulate morphological and phenotypic cell changes, more complicated interactions between cells and their heterogeneous environment as well as the impact of nutrient deficiency, for example [52,70]. These additional mechanisms could mean that the amount of data required to independently identify and quantify each mechanism *in vivo* could be impossible to collect. However, despite these difficulties, the fundamental premise of our framework, that we require additional data to uniquely identify additional mechanisms, remains valid.

We would also like to acknowledge and discuss some further difficulties that directly impacted our 2D *in vitro* assays. One of the original aims of this work was to analyse experiments over a period of *t*=72 hours to determine whether acquiring additional data over a longer period of time would assist in identifying and quantifying the mechanisms driving *in vitro* colony expansion. Unfortunately, during our initial set of experiments we observed that the cell culture medium became discoloured after *t*=72 hours, indicating that the cells were stressed. Fortunately, no such indication of cell stress was evident before *t*=72 hours, which is why we have presented data here for *t* = 0,24 and 48 hours. A preliminary analysis of the data associated with the *t*=72 hour experiments did not provide us with any additional information about the mechanisms driving colony expansion and this suggests that the difficulties associated with interpreting our data after *t*=0, 24 and 48 hours can not be alleviated by performing longer experiments. In summary, our approach is limited since we could only perform experiments over a relatively short period of time.

Originally, we also aimed to perform experiments at different initial cell densities. During our preliminary experimental investigations we found that cell colonies initialised with less than 15,000 cells produced extremely diffuse fronts that were impossible to locate and analyse using the image analysis software employed here. We also performed experiments that were initialised with more than 35,000 cells and found that these high density barrier assays tended to form 3D cell aggregates instead of a 2D monolayer. One constraint of our present modelling approach is that it is suitable for describing the expansion of 2D cell colonies and cannot be directly applied to 3D experiments [7]. These difficulties mean that our methods were restricted to a range of initial cell densities. Despite these restrictions, our systematic approach of analysing multiple data sets from the same experiment provided us with practical insights into the role of various mechanisms that drive the expansion of melanoma cell colonies. We anticipate that this approach could be used quantify the roles of cell motility, cell proliferation and cell–to–cell adhesion in different melanoma cell lines and other cell lines.

## Conclusions

In this work, we used a combined experimental and mathematical modelling approach to systematically quantify the cell motility rate, strength of cell–to–cell adhesion and cell proliferation rate in an expanding colony of MM127 melanoma cells. Our work illustrates that the relative contributions of cell motility, cell–to–cell adhesion and cell proliferation are impossible to assess using standard experimental approaches, such as measuring the area enclosed by the leading edge. Our work highlights the importance of using multiple data types to independently identify and quantify the mechanisms involved in the spatial expansion of both melanoma cell colonies and we anticipate that our approach will also be relevant to other cell lines.

## Methods

### Cell culture

Human malignant melanoma cells (MM127, [49-51]), a gift from Mitchell Stark (Queensland Institute of Medical Research), were cultured in RPMI–1640 with 2mM L-Glutamine, 23mM HEPES (Invitrogen, Australia) with 10% foetal calf serum (FCS; Hyclone, New Zealand) and 1% v/v penicillin/streptomycin (Invitrogen, Australia) in 5% CO_2_ at 37 °C and 95% air atmosphere. Cells were harvested just prior to confluence using 0.05% trypsin–EDTA(1 ×) (Invitrogen, Australia). Cell viability was determined using a trypan blue exclusion test and cell number determine using a haemocytometer.

### Circular barrier assay

Metal–silicone barriers, 6 mm in diameter (Aix Scientifics, Germany), were cleaned, sterilised using 70% Ethanol, dried and placed in the centre of each well of a 24–well tissue culture plate. Each well has a diameter of 15.6 mm. Experiments were performed using two different cell densities: 20,000 or 30,000 cells per well. To suppress cell proliferation, 10 *μ*gmL^−1^ Mitomycin–C (Sigma Aldrich, Australia) was added to half of all cell solutions for one hour at 37°C prior to transfer to the wells [60]. 100 *μ*L of cell suspension was carefully inserted into the barrier to ensure that the cells were approximately evenly distributed. Cells were allowed to settle and attach for four hours in a humidified incubator at 37°C, 5% CO_2_ and 95% air atmosphere. Assays commenced with the removal of the barrier and the cell layer was washed with warm serum free medium (SFM; culture medium without FCS) and replaced with 0.5 mL of culture medium. Cultures were incubated at 37°C in 5% CO_2_ and 95% air atmosphere for *t*=0, 24 and 48 hours. Each assay, for each time point, was repeated three times.

### Detection of motility and cell–to–cell adhesion proteins in MM127 cells using immunofluorescence and western blotting

The presence of mesenchymal-associated proteins including vimentin, N–cadherin and the epithelial-associated protein E–cadherin were demonstrated with immunofluorescence. Circular barrier assays were repeated on coverslips with 30,000 cells, for *t*=48 hours, without Mityomycin–C pretreatment. Cells were fixed with 10% neutral buffered formalin, permeabilised with 0.1% Triton X–100 in PBS for 10 minutes, blocked with 0.5% BSA in PBS for 10 minutes and incubated with a primary antibody for 90 minutes. The secondary antibody was then added to the cells for 60 minutes. Between each stage, cells were washed three times with 0.5% BSA and five times after the addition of the secondary antibody. Images were acquired using a Leica SP5 confocal microscope fitted with a Nikon digital camera. The primary antibodies were as follows; Vimentin (Monoclonal, rabbit anti–human; clone SP20, Thermo Fisher LabVision, Australia), N–cadherin (Monoclonal, Mouse anti–human, clone 32, BD Transduction Laboratories, Australia) and E–cadherin (Monoclonal, mouse anti–human, clone HECD–1, Abcam, Australia). The secondary antibodies were as follows; Vimentin (Goat anti–rabbit, Alexa Fluor–568, Invitrogen, Australia) and for both N–cadherin and E–cadherin (Goat anti–mouse, Alexa Fluor–488, Invitrogen, Australia). Western blot was also performed to confirm that E–cadherin is not expressed in MM127 cells. The same primary antibody was used as in the immunofluorescence testing, while the secondary antibody used was (goat anti–mouse, Horseradish Peroxidase Conjugate, Invitrogen, Australia). Highly adhesive breast cancer cells (MCF–7, ATCC, Manassas, VA) were used as a positive control for E–cadherin immunoreactivity [71].

### Image acquisition and analysis

Colony–scale images to show the spatial expansion of the cell colonies were obtained by fixing cells with 10% neutral buffered formalin, followed by 0.01% crystal violet (Sigma-Aldrich, Australia) in 0.1 M borate buffer. The stain was rinsed with phosphate–buffered saline (Invitrogen, Australia) and samples air–dried. Images were acquired using a stereo microscope fitted with a Nikon digital camera. Images were analysed using customised software written with MATLAB’s Image Processing Toolbox (v7.12) [72]. Edge detection and segmentation algorithms were applied to the colony–scale images to identify and isolate the entire cell colony from the background of the image, resulting in an estimate of the location of the leading edge [see Additional file 1] [7,62].

Individual–scale images detailing the number and location of the cells in the colony were acquired by destaining the crystal violet stained samples with 70% ice–cold ethanol and staining the nuclei with 1 mgml^−1^ Propidium Iodide (Invitrogen, Australia) in PBS. Images were acquired using a Nikon Eclipse Ti inverted microscope fitted with a Nikon digital camera. Overlapping adjacent images were used to reconstruct horizontal and vertical transects through the entire colony. Images were analysed using customised software written with MATLAB’s Image Processing Toolbox (v7.12) [72]. Segmentation algorithms were used to automatically count the number of cells in the Propidium Iodide stained images[7]. For some images, we found that a number of cells had to be manually identified and counted. In all cases, a visual check was performed to validate that all cells had been identified correctly using the software, or through manual counting [see Additional file 1].

### Assessing goodness of fit

To assess the goodness of fit for each type of data, we calculated the least squares error between the experimental measurements and the corresponding measurements from the model simulations.

#### Data 1: Location of the leading edge

For each set of *D* and *q* combinations tested in the main manuscript, the least squares error was calculated by comparing the average radius of the experimental expanding cell colony, *Er*_*i*_, and the average radius of the simulated expanding cell colony, *Sr*_*i*_ given by,

(3) $${\text{Error}}_{\text{LE}}(D,q)=\frac{\overset{2}{\underset{i=1}{\sum }}{\left({\text{Er}}_{i}-{\text{Sr}}_{i}\right)}^{2}}{\overset{2}{\underset{i=1}{\sum }}{\left({\text{Er}}_{i}\right)}^{2}},$$

where, *i* corresponds to the two time points, *t*=24 and *t*=48 hours. In all cases, *Er*_*i*_ and *Sr*_*i*_ correspond to the average of three experimental and three simulation replicates.

#### Data 2: Cell density profiles

The least squares error, Error_*DP*_, for each set of *D* and *q* parameter sets was calculated by comparing the averaged simulated cell density profile and the corresponding averaged experimental profile using,

(4) $${\text{Error}}_{\text{DP}}(D,q)=\frac{\overset{2}{\underset{i=1}{\sum }}\left(\overset{98}{\underset{j=1}{\sum }}{\left({\text{Ed}}_{i}^{j}-{\text{Sd}}_{i}^{j}\right)}^{2}\right)}{\overset{2}{\underset{i=1}{\sum }}\left(\overset{98}{\underset{j=1}{\sum }}{\left({\text{Ed}}_{i}^{j}\right)}^{2}\right)}.$$

Here, ${\text{Ed}}_{i}^{j}$ corresponds to the averaged non–dimensional cell density of the *j*^th^ section of the cell density profile at time *i*, where *i* corresponds to the two time points, *t*=24 and *t*=48 hours, averaged using three replicate experimental cell density profiles. Similarly, ${\text{Sd}}_{i}^{j}$ corresponds to the equivalent density of the simulated cell density profiles, averaged over five realisations.

#### Data 3: Degree of cell clustering

To determine the optimal value of *q*, the least squares error, Error_*C*_, was calculated by comparing the average proportion of isolated cells in the experiments to the model simulations. The least squares error is given by,

(5) $${\text{Error}}_{C}(D,q)=\frac{\overset{2}{\underset{i=1}{\sum }}{\left({\text{Ec}}_{i}-{\text{Sc}}_{i}\right)}^{2}}{\overset{2}{\underset{i=1}{\sum }}{\left({\text{Ec}}_{i}\right)}^{2}},$$

where, *Ec*_*i*_ corresponds to the proportion of cells clustered averaged over six replicates in the experiments, *Sc*_*i*_ corresponds to the proportion of isolated simulated cells in the model simulations, averaged over twenty realisations and *i* corresponds to the two time points, *t*=24 and *t*=48 hours.

#### Data 4: Cell density counts

To estimate *λ*, we found the value of *λ* that minimised the least squares error between our experiments and the solution of the logistic equation. Here the least squares error is given by,

(6) $${\text{Error}}_{P}(\lambda )=\frac{\overset{2}{\underset{i=1}{\sum }}{\left({\text{Ep}}_{i}-{\text{Sp}}_{i}\right)}^{2}}{\overset{2}{\underset{i=1}{\sum }}{\left({\text{Ep}}_{i}\right)}^{2}},$$

where, *Ep*_*i*_ corresponds to the non–dimensional cell density averaged over four experimental replicates, *Sp*_*i*_ is the corresponding non–dimensional cell density from the solution of the logistic equation and *i* corresponds to the two time points, *t*=24 and *t*=48 hours.

## Competing interests

The authors declare that they have no competing interests.

## Authors’ contributions

KKT, MJS and DLSM conceived the study and designed the experiments. KKT and PH performed the experiments. KKT analysed the data. KKT and MJS wrote the manuscript. KKT, MJS, KJM, DIL, DLSM and REB revised the paper. All authors read and approved the final manuscript.

## Supplementary Material

Additional file 1 **Supplementary material.** Supplementary material includes experimental data, additional figures and image analysis details. Click here for file
