# Supplementary material for: Multiple types of data are required to identify the mechanisms influencing the spatial expansion of melanoma cell colonies
Source: BMC Syst Biol. 2013 Dec 12;7:137. doi: 10.1186/1752-0509-7-137 (PMC3878834; doi:10.1186/1752-0509-7-137)
Supplement: Additional file 1 — Supplementary material. Supplementary material includes experimental data, additional figures and image analysis details. [file 1752-0509-7-137-S1.pdf]

Additional File 1 for 'Multiple types of data are required to identify the mechanisms influencing the spatial expansion of melanoma cell colonies'

Katrina K Treloar[1,2], Matthew J Simpson*[1,2], Parvathi Haridas[2], Kerry J Manton[2], David I Leavesley[2], DL Sean McElwain[2] and Ruth E Baker[3]

[1] Mathematical Sciences, Queensland University of Technology, Brisbane, Australia
[2] Tissue Repair and Regeneration Program, Institute of Health and Biomedical Innovation, Queensland University of Technology, Brisbane, Australia
[3] Centre for Mathematical Biology, Mathematical Institute, Radcliffe Observatory Quarter, Woodstock Road, Oxford, OX2 6GG United Kingdom

Email: Matthew J Simpson*- matthew.simpson@qut.edu.au;

*Corresponding author

Contents

1 Estimating the diameter of the cell nucleus **2**

2 Data type 1: Location of the leading edge **3**

3 Data type 2: Cell density profiles **4**

4 Data type 3: Degree of cell clustering **5**

5 Data type 4: Cell density counts **6**

6 Predicting the spatial expansion of a MM127 melanoma cell colony **7**

7 Image acquisition and analysis **8**

1 Estimating the diameter of the cell nucleus

High magnification images of MM127 cells were used to obtain an estimate of the mean diameter of the cell nucleus. Images were acquired using a Nikon TI Eclipse microscope fitted with a Nikon digital camera. ImageJ was used to measure the diameter of the cell nucleus in the images (Figure 1). These measurements are reported in Table 1, and indicate that the mean diameter of the MM127 cell nucleus is approximately 18 μm.

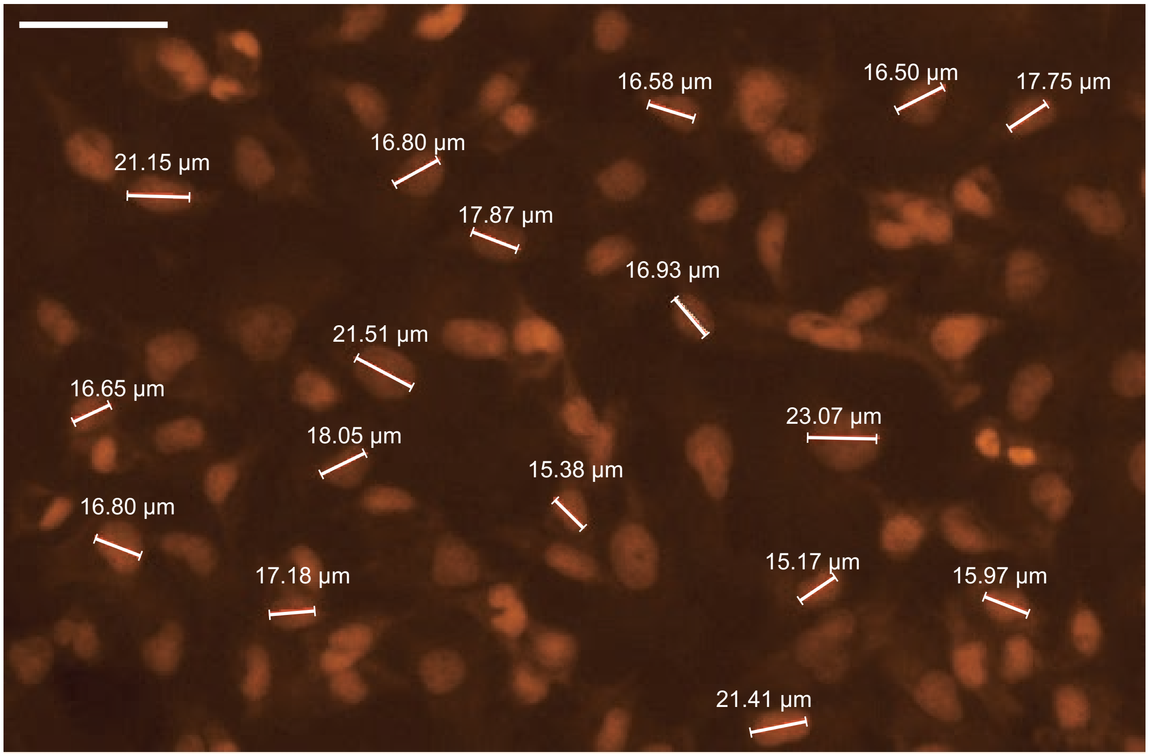

Figure 1: High magnification images of MM127 cells. Images were acquired using a Nikon Ti Eclipse microscope fitted with a Nikon digital camera and the diameter of each cell nucleus was measured using ImageJ software. The scale bar corresponds to 50 μm

Diameter of cell nucleus (μm)	18.94	18.78	21.98	17.34	23.50	17.00	16.00	16.74	16.42	15.03
	16.49	18.95	17.00	21.52	16.15	16.86	16.11	14.17	19.32	21.15
	16.80	17.87	16.58	16.50	17.75	16.93	16.65	16.80	21.51	18.05
	17.18	15.38	23.07	15.17	15.97	21.41	22.48	17.58	16.50	18.00
Mean (μm)	17.94									
Standard deviation (μm)	2.37									

Table 1: Cell diameter measurements of 40 MM127 cells indicate that the mean diameter of the cell nucleus is 17.94 ± 2.37 μm

2 Data type 1: Location of the leading edge

Image analysis software was used to detect the location of the leading edge of the expanding MM127 cell colonies. All measurements of the location of the leading edge were converted to an equivalent circular radius R. Table 2 summarises the leading edge data for all experiments performed. All measurements reported are averaged over three experimental replicates.

Initial number of cells	Time (hours)	R (Motility and adhesion) Mean (mm)	R (Motility and adhesion) Standard deviation (mm)	R (Motility, adhesion and proliferation) Mean (mm)	R (Motility, adhesion and proliferation) Standard deviation (mm)
20,000	0	3.2476	0.0223	3.2476	0.0223
	24	3.3007	0.0250	3.3407	0.0206
	48	3.3633	0.0305	3.4672	0.0506
30,000	0	3.2583	0.0208	3.2583	0.0208
	24	3.3277	0.0292	3.3807	0.0517
	48	3.3644	0.0198	3.6143	0.0229

Table 2: Experimental radius measurements of the entire expanding cell colonies for all experiments performed. Image processing was used to determine the area of the expanding colony for each experiment with and without Mitomycin–C pretreatment at $t = 0$, $t = 24$ and $t = 48$ hours for both initial densities. The area of the expanding colony was converted into an equivalent circle from which we estimated the radius $R = \sqrt{A/\pi}$. Each data point was replicated three times to give the mean radius and standard deviation.

3 Data type 2: Cell density profiles

Cell density profiles were extracted from Propidium Iodide stained images which show the location of the nucleus of individual cells throughout the entire colony. Cell density profiles for each experiment were averaged over three experimental replicates as described in the main manuscript [see section *Data 2: Cell density profiles*]. Figure 2 compares the cell density profiles extracted from three replicate experiments with the final averaged cell density profile for experiments initialised with 20,000 and 30,000 cells both with and without Mitomycin–C pretreatment. For all experiments, the averaged cell density profile appears to be an appropriate approximation given that the variation between the three replicate cell density profiles is minimal.

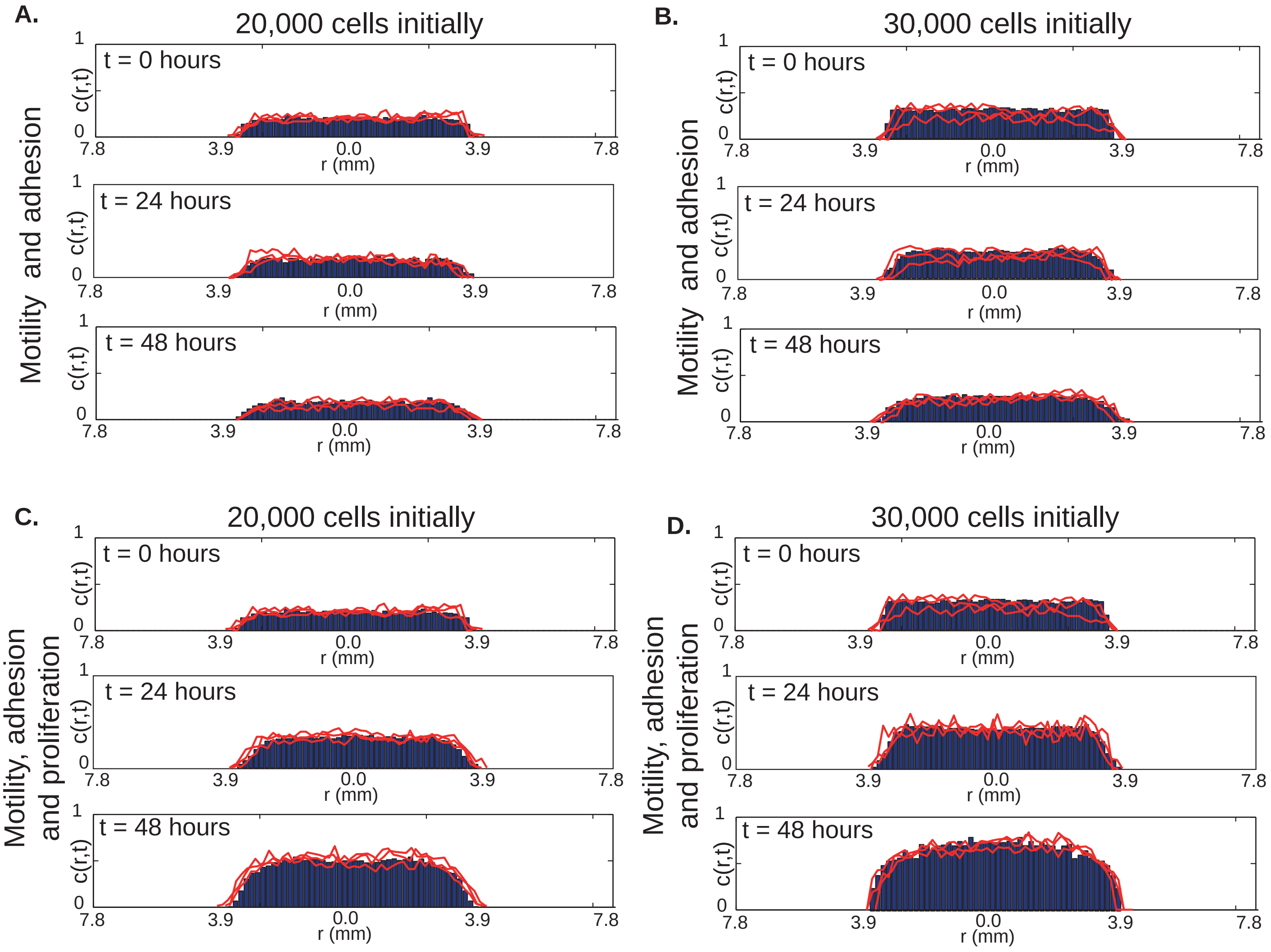

Figure 2: Extracted cell density profiles (red lines) are compared with the averaged cell density profile (blue histograms) for experiments initialised with 20,000 cells and 30,000 cells, with and without Mitomycin–C pretreatment at $t = 0$, $t = 24$ and $t = 48$ hours. Cell density profiles were averaged as described in the main text. Results in (**A**–**B**) correspond to experiments with Mitomycin–C pretreatment, while results in (**C**–**D**) correspond to experiments without Mitomycin–C pretreatment. For each experiment, the red lines correspond to the cell density profile extracted from each replicate experiment.

4 Data type 3: Degree of cell clustering

The degree of cell clustering in the MM127 cell colonies was measured by counting the number of isolated cells in Propidium Iodide images showing the location of the nucleus of individual cells throughout the entire colony. Table 3 summarises the proportion of isolated cells compared to total number of cells in six subregions in the middle of the colony as described in the main manuscript.

Initial number of cells	Time	% Isolated cells in the colony	Mean	Standard deviation
20,000	0	34.14 36.01 45.25 33.68 36.50 47.19	38.80	5.88
	24	36.01 34.14 37.65 34.25 41.58 32.25	35.98	3.30
	48	29.82 37.17 33.68 31.81 28.58 36.71	32.96	3.54
30,000	0	15.69 13.83 11.25 15.23 16.98 18.25	22.58	5.01
	24	15.69 13.83 11.25 15.23 16.98 18.25	15.21	2.46
	48	11.96 12.58 9.94 11.9 10.88 12.58	11.64	1.04

Table 3: Proportion of isolated cells in the MM127 cell colonies with Mitomycin–C pretreatment. Image processing was used to identify the number of isolated cells and the total number of cells in the expanding colony for each experiment with Mitomycin–C pretreatment at $t = 0$, $t = 24$ and $t = 48$ hours. The proportion of isolated cells in the expanding colony was converted into a percentage. Each data point was replicated six times to give the mean and standard deviation.

5 Data type 4: Cell density counts

The rate of cell proliferation in the cell population was quantified by counting the number of cells in four subregions located in the centre of the cell colonies for each experiment and at each time point. Results in Table 4 summarise the nondimensional cell density measurements. The proliferation rate, λ, was estimated by comparing the time evolution of the experimental non–dimensional cell density measurements with the corresponding solutions of the logistic equation for various values of λ as described in the main manuscript [see section *Data 4: Cell density counts*]. An estimate of the least squares error was used to determine the optimal value of λ for each experiment [see methods *Assessing goodness of fit*]. Results in Figure 3 illustrate the corresponding error, $\text{Error}_P(\lambda)$ for different values of λ for experiments without Mitomycin–C pretreatment. For each initial cell density, we observe a well–defined minimum, indicating that the proliferation rate is $\lambda = 0.0305$ hours^{-1} for experiments initialised with 20,000 cells and $\lambda = 0.0398$ hours^{-1} for experiments initialised with 30,000 cells.

Initial number of cells	Time (hours)	Motility and adhesion (with Mitomycin–C) c(t) (cells μm^{-2})	Mean	Standard Deviation	Motility, adhesion and proliferation (without Mitomycin–C) c(t) (cells μm^{-2})	Mean	Standard Deviation
20,000	0	0.2015 0.2058 0.1958 0.2158	0.2047	0.0084	0.1757 0.1909 0.2100 0.1871	0.1909	0.0149
	24	0.1985 0.2150 0.2048 0.2008	0.2048	0.0073	0.3399 0.3322 0.3513 0.3590	0.3456	0.0119
	48	0.1985 0.2058 0.2150 0.2058	0.2063	0.0084	0.5227 0.4769 0.4616 0.5303	0.4979	0.0338
30,000	0	0.2750 0.2993 0.3150 0.3447	0.3085	0.0292	0.2688 0.3421 0.2750 0.3054	0.2979	0.0336
	24	0.2627 0.323 0.3090 0.3269	0.3056	0.0300	0.5993 0.5814 0.5695 0.6706	0.6052	0.0453
	48	0.2627 0.3055 0.2912 0.3387	0.2995	0.0316	0.7251 0.7213 0.6869 0.6220	0.6888	0.0477

Table 4: Experimental measurements of the non–dimensional cell density, $c(t)$. Image processing was used to count the total number of cells in four subregions located in the centre of the cell colonies for each set of experiments, with and without Mitomycin–C pretreatment. The number of cells was converted into a non–dimensional cell density. Each data point was replicated four times to give the mean non–dimensional cell density and standard deviation.

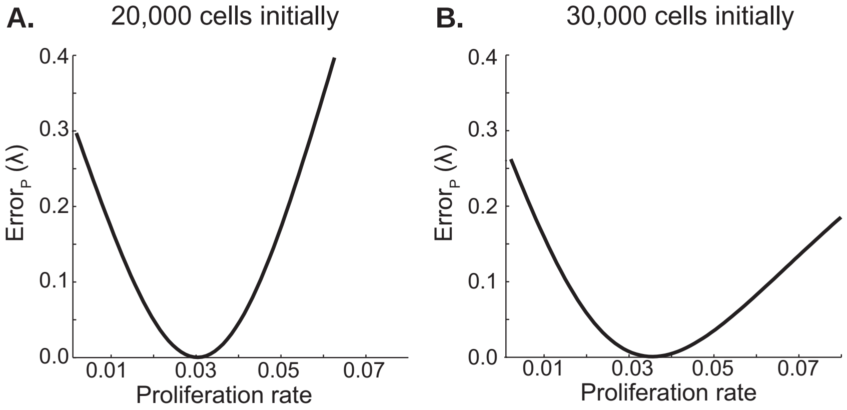

Figure 3: An estimate of the least squares error was used to determine the proliferation rate in the MM127 melanoma cell colony. (**A**–**B**) indicates the error, $\text{Error}_P(\lambda)$ produced for various values of λ between 0.001 and 0.08 for experiments initialised with 20,000 and 30,000 cells, respectively.

6 Predicting the spatial expansion of a MM127 melanoma cell colony

Table 5 summarises the estimates of the cell diffusivity, D, cell–to–cell adhesion strength, q, and cell proliferation rate, λ, obtained from the analysis described in the main manuscript.

Initial number of cells	Cell diffusivity (D)	Cell–to–cell adhesion strength (q)	Proliferation rate (λ)
20,000	162 μm^2 hour^{-1}	0.3	0.0305 hour^{-1}
30,000	243 μm^2 hour^{-1}	0.5	0.0398 hour^{-1}

Table 5: Estimates of the cell diffusivity, D, cell–to–cell adhesion strength, q and cell proliferation rate λ obtained from the analysis described in the main manuscript.

Experimental images of the entire expanding cell colony and the corresponding simulated cell colonies using the estimates of D, q and λ are shown in Figure 4. The location of the leading edge and the radius of the expanding cell colonies are superimposed on both experimental and model images of the colony. In all cases, the estimates obtained using the analysis described in the main manuscript visually appear to predict the location of the leading edge of the MM127 cell colonies.

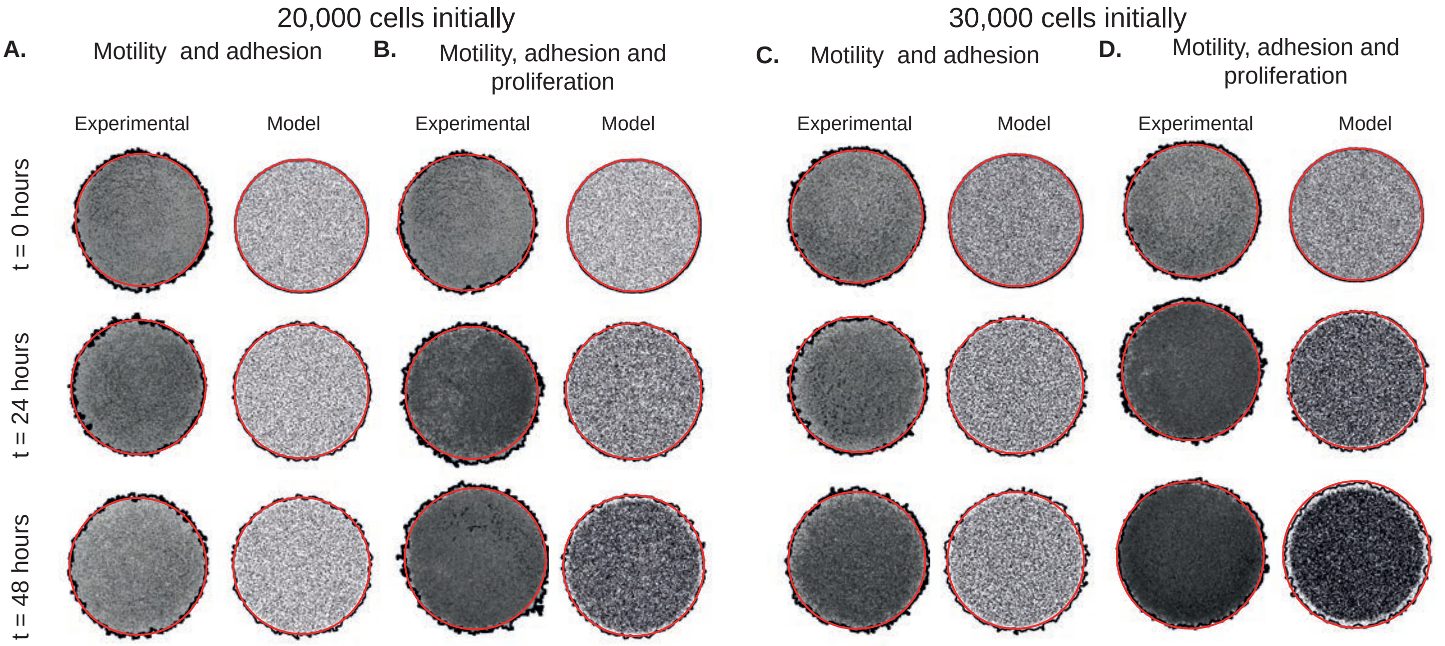

Figure 4: (**A**–**D**) Experimental images of the entire expanding cell colony for each different set of experiments and corresponding model simulations using the parameter estimates obtained in the manuscript. In all images, the detected location of the leading edge is outlined in black while the equivalent mean radius of the expanding colony is shown in red. Model simulations of the experiments initialised with $20,000$ cells were performed using $D = 162$ μm^2 hour^{-1}, $q = 0.3$ and $\lambda = 0.0305$ hour^{-1}, and for experiments initialised with $30,000$ cells, simulations were performed using $D = 243$ μm^2 hour^{-1}, $q = 0.5$ and $\lambda = 0.0398$ hour^{-1}.

7 Image acquisition and analysis

Detecting the location of the leading edge of the cell colony

Customised image processing software was written in MATLAB's image processing toolbox. The same software was used to detect the location of the leading edge in both the experimental cell colonies and the simulated cell colonies. Each colour image was imported (*imread*) and converted to greyscale (*rgbtogray*). A binary gradient mask containing the segmented cell colony was obtained by applying the Sobel operator (*edge(Original Image, 'Sobel'), edge(I,'sobel',threshold*) to enhance lines of high contrast. To show the outline of the object, the lines in the binary gradient mask were dilated (*strel, imdilate*). Remaining holes in the images were filled (*imfill*) and objects disconnected from the edge were removed (*imclearborder*). The image was smoothed and filtered to remove small objects detected in the previous steps (*imerode, medfilt2*). The resulting image contains both a large object (corresponding to the expanding cell colony) and smaller objects. The smaller objects were removed (*regionprops, bwareopen*) to leave the edge of the cell colony. An outline of the detected edge was superimposed back onto the original image (*bwperim*) to verify the accuracy of the procedure. The area (*regionprops*) of the detected object was estimated and converted into an equivalent circular radius.

Detecting individual cells in the cell colony

To count the number of cells in the various subregions, we used a combination of customised image processing software, written using the MATLAB image processing toolbox, and manual counting where necessary. Each colour image was imported (*imread*), converted to greyscale (*rgbtogray*) and enhanced (*imadjust*) to provide sufficient contrast between each cell and the background of the image. The image was converted to black and white based on a threshold (*graythresh, im2bw*). To reduce noise, objects less than 30 pixels were removed (*bwareaopen*). Remaining holes in the image were filled (*strel, imfill, Bwboundaries*), using a similar method as in the leading edge software. The centre of each detected region (which we assume to be an individual cell) was identified (*regionprops(image,'Centroid')*) and superimposed back on the original image to test the accuracy of the detection method. The number of cells detected by the automated software was recorded. All remaining cells not automatically identified were manually included in the total cell count.

Identifying isolated cells in the cell colony

In addition to counting individual cells, we identified isolated cells that did not share a circular region, of radius 18 μm, with other cells. To do this, we repeated the same image processing procedure to identify the total number of cells in the colony. For each identified region corresponding to a cell, we recorded the physical location of each identified cell using (*regionprops*). Each identified cell was checked to determine whether the cell was isolated by comparing the location of the identified cell with the locations of all other cells. For example, to check if cell A, located at (x_1, y_1), and cell B, located at (x_2, x_2) share the same circular region of radius 18 μm, we calculated the physical distance between the two cells using $W = \sqrt{(x_2 - x_1)^2 + (y_2 - y_1)^2}$. If $W > 18$ μm, this indicates that cell B does not share the same circular region of radius 18 μm around cell A and vice versa. This was repeated systematically for all cells to identify which cells were completely isolated in the cell colony. To test the accuracy of the detection method, we superimposed the locations of each isolated cell

back onto the original image and overlaid a square grid of size 18 μm. The image was visually checked to make sure all identified isolated cells were correctly detected and that the image processing had identified all isolated cells. In some cases, a small number of identified cells were incorrectly identified and were deleted. Similarly, a small number of isolated cells were not identified and had to be manually added.
